# ALDH5A1/miR-210 axis plays a key role in reprogramming cellular metabolism and has a significant correlation with glioblastoma patient survival

**DOI:** 10.1186/s12935-024-03432-z

**Published:** 2024-07-22

**Authors:** Indranil Mondal, Neelam Gupta, Vikas Sharma, Chitra Sarkar, Durga Prasad Mishra, Ritu Kulshreshtha

**Affiliations:** 1 Department of Biochemical Engineering and Biotechnology, Indian Institute of Technology Delhi, New Delhi, 110016 India; 2 Division of Endocrinology, CSIR-Central Drug Research Institute, Lucknow, 226031 India; 3 Academy of Scientific and Innovative Research, Ghaziabad, Uttar Pradesh 201002 India; 4 Centralized Core Research Facility, All India Institute of Medical Sciences, New Delhi, 110029 India; 5 Department of Pathology, All India Institute of Medical Sciences, New Delhi, 110029 India

**Keywords:** Glioblastoma, Glycolysis, OXPHOS, microRNA, Hypoxia, Metabolism

## Abstract

**Background:**

Glioblastoma (GBM) is the most aggressive among the tumors of the central nervous system (CNS), and has a dismal prognosis. Altered metabolism, especially the increased rate of aerobic glycolysis promotes rapid proliferation of GBM cells. Here, we investigated the role of aldehyde dehydrogenase 5 family member A1 (ALDH5A1), a mitochondrial enzyme in the aspect of GBM metabolism. We also studied the regulatory mechanisms of altered ALDH5A1 expression in GBM.

**Approach and results:**

We show that ALDH5A1 is significantly downregulated in GBM patients in a grade dependent manner as compared to control brain and its low expression is associated with poor prognosis. It is significantly downregulated under hypoxia and is a direct target of the hypoxia induced microRNA: miR-210. Ectopic overexpression of ALDH5A1 in GBM cell lines U-87 MG and T98G markedly reduced their proliferation, 3D spheroid forming ability, and formation of reactive oxygen species (ROS). ALDH5A1 upregulation increased the oxygen consumption rate (OCR), and reduced the extracellular acidification rate (ECAR) of GBM cells while miR-210 overexpression showed the opposite. A significant downregulation in the transcript levels of LDHA, PDK1, and SLC2A1; coupled with lower glucose uptake and lactate production upon ALDH5A1 overexpression reveals that ALDH5A1 significantly reduces the glycolytic capacity of GBM cells. Total ATP generated in 24 h was more when miR-210 was overexpressed, while a slight decrease in ATP formation was observed upon ALDH5A1 upregulation. Interestingly, we also observed that ALDH5A1 expression is elevated and miR-210 levels are downregulated in IDH-mutant glioma as compared to its wild-type form.

**Conclusion:**

Overall, our findings suggest that miR-210 mediated downregulation of ALDH5A1 plays a critical role in tumor metabolism and helps maintaining a high glycolytic phenotype in GBM.

**Supplementary Information:**

The online version contains supplementary material available at 10.1186/s12935-024-03432-z.

## Introduction

Glioblastoma (GBM) is the most aggressive and lethal tumor of the central nervous system (CNS), having a dismal prognosis. Patients suffering from GBM have a median overall survival (OS) of approximately 15 months only [1]. GBM is usually characterized by its highly invasive nature, therapy resistance, stem cells rich hypoxic core, intra/inter-tumoral heterogeneity, and an immunosuppressive microenvironment. The current treatment modality is limited to maximal safe surgical resection, followed by radio and chemotherapy.

During the past decade, a growing body of evidence has suggested that altered metabolism is a major hallmark of cancer that can be targeted for effective treatment of various malignancies such as GBM, breast cancer, and colon cancer. Similar to many other cancers, GBM cells rely heavily on aerobic glycolysis (also known as the Warburg effect), where, even in the presence of oxygen, cells readily undergo glycolysis instead of oxidative phosphorylation for rapid turnover of energy, and other building blocks of the cell such as nucleotides and lipids [2]. A recent study by Stanke et al. revealed that high expression of the two key glycolytic genes HK2 and PKM2, along with low expression of SDHB and COX5A- two important enzymes of mitochondrial respiration, was associated with poor OS of GBM patients [3]. Thus, the metabolic rewiring in GBM is clinically relevant, and more in-depth studies are required to discover novel players of GBM metabolism.

Here we focus on succinic semialdehyde dehydrogenase (SSADH), a mitochondrial protein encoded by the nuclear gene aldehyde dehydrogenase 5 family member A1 (ALDH5A1). We study its expression, regulation and functional role in GBM, while emphasizing on tumor metabolism. ALDH5A1 belongs to the aldehyde dehydrogenase gene superfamily, members of which primarily carry out enzymatic detoxification of reactive aldehydes along with the biosynthesis of various important biomolecules [4]. The primary role of ALDH5A1 is the metabolism of the inhibitory neurotransmitter gamma-aminobutyric acid (GABA) and its deficiency causes a rare autosomal recessive disorder known as SSADH deficiency. GABA metabolism is closely linked to the TCA cycle via the metabolites alpha-ketoglutarate and succinate. GABA is converted to succinic semialdehyde (SSA) by the enzyme GABA transaminase and this SSA is further catalyzed by SSADH, producing succinate which is ultimately fed to the TCA cycle. This auxiliary process which supplies biomolecules to the TCA cycle is also known as the GABA shunt. Interestingly, molecular bioenergetics studies on an ALDH5A1-/- mouse model revealed mitochondrial dysfunction in the hippocampus where the activities of the electron transport chain (ETC) complexes I-IV got diminished [5]. Thus, dysregulation of ALDH5A1 levels would most likely alter cancer cell metabolism, by disturbing the balance between the major metabolic pathways- oxidative phosphorylation and aerobic glycolysis. As reports on the role on ALDH5A1 in cancer metabolism are scarce, we were interested to study its functional role in GBM metabolism.

Our findings suggest that ALDH5A1 is heavily downregulated in GBM as compared to the normal brain. Its expression decreases under hypoxic conditions and is post-transcriptionally regulated by miR-210, an oncogenic microRNA in GBM. Interestingly, miR-210 has been attributed previously with mitochondrial dysfunction. When ALDH5A1 was overexpressed in GBM cells, we observed increased oxygen consumption rate (OCR) and diminished extracellular acidification rate (ECAR), suggesting high mitochondrial activity and decreased glycolysis. This observation was supported by a decrease in glucose uptake and extracellular lactate formation. MiR-210 overexpression revealed opposite results. Moreover, ALDH5A1 overexpression decreased cellular proliferation, 3D-spheroid forming ability, and the formation of reactive oxygen species (ROS) of GBM cells, suggesting its tumor suppressive role. Overall, this study reports for the first time the role of ALDH5A1 in reshaping the metabolic scenario of GBM cells, and suggests that miR-210 mediated downregulation of ALDH5A1 might help GBM cells to maintain a high glycolytic phenotype.

## Materials and methods

### Public data acquisition

The GBM patient data was analyzed using various public datasets such as The Cancer Genome Atlas (TCGA: https://www.betastasis.com/glioma/tcga_gbm/), Chinese Glioma Genome Atlas (CGGA: http://www.cgga.org.cn/), GlioVis (http://gliovis.bioinfo.cnio.es), Gene Expression Profiling Interactive Analysis (GEPIA2: http://gepia2.cancer-pku.cn/#index), and The Human Protein Atlas (THPA: https://www.proteinatlas.org/). Mutation data of ALDH5A1 in GBM patients was obtained from the cBioPortal (https://www.cbioportal.org/) database.

### Patient samples

This study utilized GBM samples collected during surgery at the All-India Institute of Medical Sciences (AIIMS), Delhi. The Institute Ethics Committee approved the study, and it was conducted according to ethical standards. Tumor samples collected were quickly frozen in liquid nitrogen and stored at − 80 ºC until further use. All tumor cases had classical histological features of GBM namely anaplasia, frequent mitosis, necrosis and microvascular proliferation. Immunohistochemistry for IDH1R132H mutation was available in 18 of these cases and all cases were IDH1 wild type. RNA was extracted from selected 33 GBM samples and 3 epileptic brain samples (representing non-cancerous brain tissue) using the RNeasy mini kit (Qiagen, USA) and was quantified with the Nanodrop instrument. The iScirpt kit (Bio-Rad, USA) was used to synthesize cDNA from 500 ng of RNA from each sample. The levels of ALDH5A1 were measured in the samples using SyBr Green (Bio-Rad, USA) with GAPDH as the internal control. The double delta CT method was used to calculate the relative fold change, and the data was compared to the control. Follow up data was available for 12 cases up to 3 years period. During this period, two patients exhibited adverse events (death), while three demonstrated progressive diseases (PD) while the remaining seven patients maintained No evidence of disease (NED).

### Anti-miR oligos, normal brain RNA and recombinant plasmids

The synthetic miRNA inhibitor to miR-210 (MISSION^®^ Synthetic microRNA Inhibitor, cat no: HSTUD0373p) and its negative control (MISSION^®^ Synthetic microRNA Inhibitor, Negative control 2, cat no: NCSTUD002) were procured from Sigma-Aldrich (St Louis, USA). Normal human brain total RNA was procured from Takara Bio (Kusatsu, Japan). The precursor sequence of miR-210 with 200 bp flanking sequence upstream and downstream (total 576 bp) cloned in the EcoRI restriction site of pBABE-Puro vector (Cell Biolabs Inc, USA) was prepared as described by Crosby et al. [6]. The coding sequence of ALDH5A1 (1608 bp) was cloned between the restriction sites of HindIII and XhoI in the mammalian expression vector pcDNA 3.1 (+) (Invitrogen, USA) as a part of this study. The 3’ UTR of ALDH5A1 containing miR-210 binding site and its flanking sequence (position 1131–1379 (249) bp of ALDH5A1 3’ UTR) was cloned between the SpeI and SacI restriction sites of the pMIR-REPORT™ Luciferase vector (Ambion^®^, USA). The pcDNA3-Flag-IDH1 (IDH wild type) and pcDNA3-Flag-IDH1-R132H (IDH mutant) were kind gifts from Dr. Yue Xiong (Addgene plasmid # 62,906 and # 62,907) [7]. The primer sequences used for amplification are given in Appendix II of Supplementary file 1.

###  Cell culture

T98G (source- ATCC) cells were a kind gift from Dr. Ellora Sen, National Brain Research Centre (NBRC), Manesar, India. The human GBM cell line U-87 MG was obtained from the National Center for Cell Science (NCCS) cell repository, Pune, India. T98G and U-87 MG cell lines were maintained in Dulbecco’s Modified Eagle Medium (DMEM, GIBCO) supplemented with 10% Fetal Bovine Serum (FBS, GIBCO), 100 U/ml penicillin, and 100 µg/ml streptomycin. The cells were grown at 37 ºC, and 5% CO2 in a humidified incubator.

### Transient transfection

Cells were seeded in a way so that they became 70–80% confluent approximately 24 h post-seeding. Briefly, about 1 × 10^5^ and 2 × 10^5^ cells were seeded in each well of a 12-well and 6-well plate, respectively. About 1.5 µg plasmid DNA/well (for each well of a 12-well plate) was complexed with Lipofectamine 2000 (Invitrogen, USA) for transfection according to the manufacturer’s protocol. For a 6-well plate, 3 µg plasmid DNA was used per well. On the other hand, 30 nM anti-miR-210 (Sigma Aldrich, USA) was used per well for miR-210 knockdown studies. 48 h post-transection, the cells were harvested for RNA/protein isolation for further cellular assays.

### Hypoxia treatment

U-87 MG and T98G GBM cells were maintained under chronic hypoxia (0.2% O_2_) for 48 h in a Ruskinn InvivO_2_ hypoxia workstation. A temperature of 37 ºC and 5% CO_2_ was maintained inside the incubator chamber. Following incubation period, cells were carefully lysed for RNA or protein isolation, maintaining minimum exposure to atmospheric oxygen.

### RNA isolation and RT-qPCR

To obtain RNA from transfected/treated cells, the RNeasy mini kit (Qiagen, USA) was used according to the manufacturer’s instructions. Briefly, about 500 ng of RNA was then reverse transcribed using the iscript cDNA synthesis kit (Bio-Rad, USA). The resulting cDNA was amplified for specific genes using the Eva Green mastermix kit (Bio-Rad, USA) along with corresponding primers (Appendix 1, Supplementary File 1) in a Bio-Rad CFX-96 Touch Real-Time PCR detection system (Bio-Rad, USA). To normalize gene expression data, either Glyceraldehyde 3-phosphate dehydrogenase (GAPDH) or β-actin (ACTB) was used as an endogenous control. For miRNA isolation, the miRNeasy kit (Qiagen, USA) was used. MiRNA levels were investigated using the stem-loop RT-qPCR method. RNU6B was used as an endogenous control for normalization purpose.

### Protein isolation and western blotting

For isolation of total protein, cells were transfected in 6-well plates. 48 h post-transfection, media was removed, cells were washed with cold PBS, and scraped in 200 µl RIPA lysis buffer supplemented with 1mM phenylmethylsulfonyl fluoride (PMSF) (Real-Gene, India) per well. The lysis process was carried out while keeping the samples on ice, with intermittent mild vortexing. Finally, the lysates were centrifuged at 10,000 rpm at 4 ºC for 20 min and protein was isolated by collecting the supernatant in pre-chilled eppendorf tubes.

The isolated protein was quantified using Bradford assay. About 40 µg of total protein was mixed with 4X Laemmli buffer and subjected to sodium dodecyl sulfate (SDS)-polyacrylamide gel electrophoresis (PAGE). The proteins were then transferred onto a nitrocellulose membrane using wet-transfer method at 4 °C (90 V, 1.5 h). Further, the membrane was blocked in 5% bovine serum albumin (BSA) in tris-buffered saline with 0.1% Tween 20 (TBST) for 1 h at room temperature. The membrane was then incubated with the primary antibodies anti-β-actin (1:2000, Santa Cruz Biotechnology, USA) and anti-ALDH5A1 (1:1500, Santa Cruz Biotechnology, USA) in an end to end rocker with mild rocking for overnight at 4 °C. Next day, the primary antibody solution was removed, and the membrane was washed three times in 1X TBST (10 min each wash), followed by the addition of horse radish peroxidase (HRP)-conjugated secondary antibody (Invitrogen, USA) for 1 h. Finally, the membrane was washed thrice in 1X TBST and the proteins were detected by using an ECL chemiluminiscent substrate (Bio-Rad, USA).

### MicroRNA target prediction and 3’ UTR dual luciferase assay

The 3’ UTR of ALDH5A1 containing miR-210 binding site (position 1131–1379 (249) bp) was identified using the TargetScan Human (release 8.0) miRNA target-prediction tool and cloned in the pmiReport luciferase vector downstream of the firefly luciferase gene. The primer sequences for cloning the 3’ UTR in the pMIR-REPORT™ Luciferase vector is listed in Appendix II of Supplementary file 1. A PCR-based site-directed mutagenesis (SDM) technique was employed for mutating three bases on the ALDH5A1 3′ UTR that binds with the seed region of miR-210. Briefly, 5′ACGCACA3′ was converted to 5′ACCGTCA3′. The primer sequences used for incorporating the substitution mutation are listed in Appendix III of Supplementary file 1. The wild type and mutated 3’ UTR were further transfected to U-87 MG GBM cells along with the miR-210 overexpressing construct or control (empty vector). A renilla luciferase construct was used as transfection control. 48 h post-transfection, the cells were lysed using a passive lysis buffer (Promega, USA) and luminescence was measured using a luminometer (Berthold, Germany). Finally, relative luminescence was plotted after normalization with the renilla luminescence.

### Protein-protein interaction and functional enrichment analysis

An *in silico* protein-protein interaction (PPI) study of ALDH5A1 was carried out using the STRING database (https://string-db.org/). A confidence-based cutoff of 0.7 was set for constructing the PPI network. The network was further exported to the Cytoscape software. Gene ontology analysis and functional enrichment of the biological pathways associated with ALDH5A1 and its interacting genes were carried out using the ShinyGO (http://bioinformatics.sdstate.edu/go/) web-based tool.

### Identification of CpG islands and 5-Azacytidine treatment

The presence of CpG islands in the promoter of ALDH5A1 was investigated using the online CpG island finder tool: Database of CpG islands and Analytical Tool (DBCAT: http://dbcat.cgm.ntu.edu.tw/). The global hypomethylating agent 5-azacytidine (Sigma Aldrich, USA) was reconstituted in dimethyl sulfoxide (DMSO) which was considered as vehicle control (VC) during treatment. T98G GBM cells were treated with 5 and 10 µM 5-Azacytidine. Fresh treatment was given at every 24 h and cells were lysed after 48 h for RNA isolation.

### Metabolic assays

The changes in cellular metabolism after miR-210 and ALDH5A1 overexpression were analyzed by measuring the lactate production, glucose uptake and ATP synthesis assays.

#### Glucose uptake

##### Flow cytometric assay

2-NBDG ([2-N-(7-nitrobenz-2-oxa-1,2-diaxol-4-yl) amino]-2-deoxyglucose) is a fluorescently tagged analog of fluorodeoxy-glucose (FDG) that was used to quantify glucose uptake without using radioactive tracers. U-87 MG cells were transfected with miR-210 and ALDH5A1 along with their respective control vectors and incubated for 48 h. After the completion of the treatment, the media was removed; cells were washed with 1X sterile PBS, and supplemented with no glucose media containing 100 µM 2-NBDG. The cells were further incubated at 37 °C and 5% CO_2_ in a humidified incubator for 30 min. Finally, the cells were washed with chilled 1X PBS, trypsinized, and resuspended in PBS for further analysis by flow-cytometry.

##### Colorimetric assay

GBM cells transfected with miR-210 and ALDH5A1 along with their respective empty vector controls were incubated for 48 h. A no-cell control (only media) was kept as reference for total glucose present initially. After 48 h, the spent media was collected from each well and glucose present in the cell culture supernatant was measured using a colorimetric assay according to the manufacturer’s protocol of the Chekine™ glucose assay kit (Abbkine, China), Glucose uptake was measured by subtracting the amount of glucose in treatment groups from the no-cell control. Cell number was used for normalization purpose.

#### Lactate production

The extracellular lactate production of GBM cells transfected with miR-210 and ALDH5A1 constructs (along with empty-vector controls) was measured using the Chekine™ lactate assay kit (Abbkine, China) according to the manufacturer’s protocol. Briefly, transfected cells were incubated for 48 h, after which the spent media was collected from each group for assaying, and the cells were further trypsinized and counted using a hemocytometer. The culture supernatant was further used for determining L-lactate levels using a lactate dehydrogenase based colorimetric assay. Lactate production was normalized using cell number. Finally relative lactate production was plotted.

#### ATP synthesis

The total ATP synthesized in GBM cells transfected with miR-210 and ALDH5A1 constructs (along with empty-vector controls) was measured using the CellTiter-Glo luminescence assay kit (Promega, USA) according to the manufacturer’s protocol. Briefly, transfected cells were harvested after 48 h by trypsinization, and 10 × 10^3^ cells were re-seeded in triplicate in each well of a 96-well plate. After 24 h, 100 µl of assay buffer was added to each well, and the cells were allowed to lyse by placing the plate in an orbital shaker for 2 min. The plate was further kept at room temperature for 10 min allowing the luminescence signal to stabilize. Finally, the luminescence was measured using a luminometer (Berthold, Germany). Readings were normalized with total protein content.

####  Seahorse XF-analyzer based metabolic assays

##### Mitochondrial stress test

The oxygen consumption rate (OCR) of GBM cells upon transfection with pC/ALDH5A1 and pBabe/miR-210 constructs was measured by performing the mitochondrial stress test. Briefly, the transfected cells were harvested by trypsinization, and 10 × 10^3^ transfected cells were plated in each well of a specialized 96-well plate compatible with the Seahorse XF analyzer. After 24 h incubation, the cells were injected with various modulators of mitochondrial respiration at set time points. Oligomycin (ATP synthase inhibitor that could rapidly hyperpolarize the mitochondrial membrane, thereby preventing protons passing through the complexes), FCCP (an uncoupling agent of oxidative phosphorylation, could reverse the hyperpolarized state caused by oligomycin through carrying the protons across the mitochondrial inner membrane), and Rotenone/Antimycin A (mitochondrial complexes I and III inhibitor for inhibition of mitochondrial respiration) were administered at a dose of 1µM at 20, 50 and 80 min respectively. The real time oxygen consumption rate (OCR/10 × 10^3^ cells) was quantified using an XF- 96 Extracellular Flux Analyzer (Seahorse Bioscience, Agilent, Santa Clara, CA, USA) according to the manufacturer’s protocol.

##### Glycolytic stress test

The extracellular acidification rate (ECAR) of GBM cells upon transfection with pC/ALDH5A1 and pBabe/miR-210 constructs was measured by performing the glycolytic stress test. Briefly, the transfected cells were harvested by trypsinization, and 10 × 10^3^ transfected cells were plated in each well of a specialized 96-well plate compatible with the Seahorse XF analyzer. After a 24-h incubation, the media was changed to assay medium (XF base medium DMEM supplemented with 2 mM glutamine), and cells were incubated in a non-CO_2_ incubator at 37 °C for 1 h before the assay. Glucose (10 mM), oligomycin (0.5 µM) and 2-DG (100 mM final) were diluted in the assay medium and administered at 20, 50 and 80 min respectively. The real time ECAR (mpH/min) was quantified using an XF- 96 Extracellular Flux Analyzer (Seahorse Bioscience, Agilent, Santa Clara, CA, USA) according to the manufacturer’s protocol

### ROS measurement

For measurement of reactive oxygen species (ROS), pC/ALDH5A1 transfected cells were harvested after 48 h and 10 × 10^3^ cells from each group were re-seeded in each well of a 96-well plate in triplicate. After 24 h, the media was replaced with DMEM containing 20 mM DCFDA solution and cells were incubated at 37 °C for 45 min. Finally, the DCFDA solution was replaced with 1X PBS and fluorescence was measured using a microplate reader (Ex/Em = 485/535 nm). Readings were normalized using total protein content of each well.

### MTT cell proliferation assay

To measure proliferation, cells were re-seeded in triplicate (5000 cells per well) in 96-well plates 48 h after transfection. Proliferation was assessed at various time points using the 3-(4,5-dimethylthiazole-2-yl)-2,5-diphenyl tetrazolium bromide (MTT)-based cell viability assay. Briefly, at designated time points, the culture media was replaced with 100 µl of fresh DMEM containing 1X-MTT solution, and the cells were incubated at 37 °C, 5% CO_2_ for 2 h in the dark. Finally, the MTT-containing media was removed and the formazan crystals were dissolved in dimethyl sulfoxide (DMSO). Cell viability was quantified by measuring absorbance at 595 nm. Relative proliferation was plotted by normalizing the absorbance values with day 0 readings.

### Colony formation assay

Transfected cells were harvested by trypsinization and re-seeded in triplicate (1000 cells per well) in a 6-well plate. Media was changed in every 3 days until the colonies in the control/test group had approximately 50 cells. The colonies were then gently washed with 1X PBS and fixed with 3.7% formaldehyde solution in PBS for 20 min. Further, the fixing solution was removed, washed with 1X PBS and stained with 0.5% crystal violet solution for 10 min. Finally, the staining solution was removed, washed with distilled water and allowed to dry on paper towels. Following colony counting, the number of colonies formed in cells transfected with pC and ALDH5A1 were plotted.

### 3D spheroid formation assay

Transfected cells were harvested by trypsinization after 48 h and reseeded in duplicate (10,000 cells/well) in 96-well plates pre-coated with 1% agar. Cells were allowed to come together and grow into 3D spheroids in a course of approximately 7 days until they crossed a diameter of minimum 300 μm. Spent media was replaced with fresh DMEM at every 48 h. Finally, the spheroids were imaged using a Nikon Eclipse Ti-S inverted microscope and their diameters were measured.

### Cell cycle analysis

48 h post transfection, the cells were trypsinized, washed with cold PBS and fixed by adding 70% ethanol to pelleted cells while vortexing. The fixing agent was removed by PBS wash and cells were resuspended in 200 µl FxCycle™ PI/RNase staining solution (Invitrogen, USA). Finally, the distribution of cells in various phases of the cell cycle was analyzed using a flow cytometer (BD FACSCalibur, USA). This experiment was performed in biological duplicates in the T98G cell line.

###  Statistical analysis

All in vitro experiments were performed in triplicates unless mentioned otherwise. Western blots were performed in duplicates. The statistical significance was calculated using a two-tailed student’s T test for comparing two groups. One way analysis of variance (ANOVA) was employed for calculating statistatical significance of the differences among the means of three or more independent variables. P value < 0.05 was considered statistically significant (*p > 0.01 and < 0.05, **p < 0.01, ***p < 0.001). Error bars indicate ± SD (Standard deviation). Graphs were plotted using Graphpad Prism software.

## Results

### Expression and clinicopathological association of ALDH5A1 in GBM

Using the Genotype Tissue Expression (GTEx) dataset and the Human Protein Atlas (HPA), we identified that ALDH5A1 has a considerably high expression in the human brain. Transcriptomic data from the GTEx dataset revealed that ALDH5A1 mRNA expression is quite high in the various parts of the human brain, including the brain cortex, which is a hotspot for GBM tumors (Figure S1A). The HPA transcript data of the brain also suggested that ALDH5A1 has consistently high expression (> 30 nTPM) in the different parts of the brain (Figure S1B). Furthermore, data from the HPA suggested that ALDH5A1 encoded protein SSADH is localized in the mitochondria (Figure S1C).

**Fig. 1:**
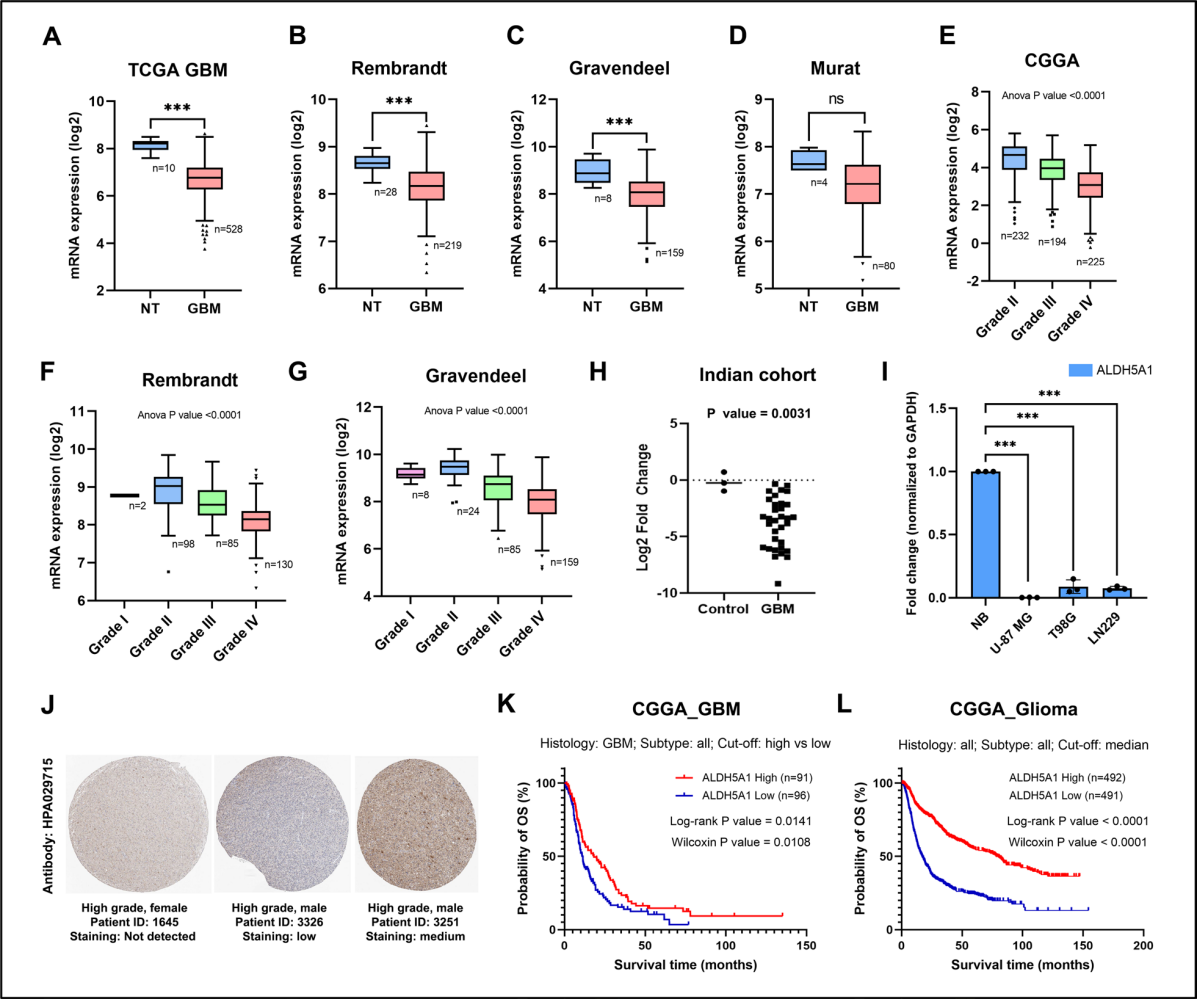
ALDH5A1 is downregulated in GBM and associated with favourable patient survival: **A–D** ALDH5A1 mRNA expression in non-tumor (NT) vs. GBM tumor patient samples. **E–G** ALDH5A1 transcript levels in various grades of glioma. **H** ALDH5A1 mRNA expression in Indian patients. **I** ALDH5A1 transcript levels in GBM cell lines and normal brain RNA. **J** Representative images of immunohistochemical staining of glioma tissue samples with HPA029715 anti-ALDH5A1 antibody. Kaplan Meier survival curve of high and low ALDH5A1 expressing GBM patients **K** and Glioma patients **L** of the CGGA database. Data was downloaded from GlioVis web portal

After observing that ALDH5A1 has high expression in the human brain, we were next interested to know whether its expression is deregulated in GBM as compared to the normal brain. For this, we downloaded ALDH5A1 mRNA expression data from various brain-tumor datasets using the GlioVis web-tool. ALDH5A1 mRNA levels were found to be significantly downregulated in the GBM tissues of the TCGA GBM, Rembrandt, and Gravendeel datasets as compared to the non-tumor samples. On the other hand, in the Murat dataset, ALDH5A1 downregulation was found to be statistically insignificant (Fig. 1A–D). We also studied the grade-wise expression of ALDH5A1 in the glioma datasets: CGGA, Rembrandt, and Gravendeel. It was found that in all three datasets, ALDH5A1 mRNA expression decreased with an increase in tumor grade, grade IV having the lowest expression (Fig. 1E–G). We further measured the transcript levels of ALDH5A1 in a cohort of Indian GBM patients (control *n* = 3, GBM *n* = 33), and found it to be significantly downregulated in Indian patients too (Fig. 1H). Finally, we confirmed this by measuring ALDH5A1 transcript levels in the GBM cell lines U-87 MG, T98G and LN229 as compared to normal brain RNA. We found significant downregulation in all three cell lines (Fig. 1I).

We were further interested to know that whether this downregulation of ALDH5A1 mRNA levels in GBM is also corroborated at the protein level as well. For this, we studied the HPA where immunohistochemical analysis of ALDH5A1 was performed in a cohort of 9 glioma patients. None of the patient tissues had high staining for ALDH5A1, while two samples had medium and one sample had low staining. Six samples did not stain for ALDH5A1 at all (Figure S1D). Figure 1J shows one of each undetected, low and medium staining of high-grade glioma tissues using HPA029715 anti-ALDH5A1 antibody. This suggested that ALDH5A1 had relatively low protein expression in glioma patient samples.

To further understand the clinical relevance of ALDH5A1 in GBM, we downloaded GBM patient survival data along with ALDH5A1 expression from various brain-tumor datasets using the GlioVis tool. Kaplan meier survival analysis was performed and it was observed that high ALDH5A1 expression is associated with better OS of GBM patients of CGGA (Fig. 1K), Gravendeel (Figure S2A), and Vital (Figure S2B), datasets. However, no statistically significant association of ALDH5A1 expression and patient survival was observed in the TCGA GBM dataset (Figure S2C). Interestingly, we found that high ALDH5A1 showed a significant correlation to better survival in multiple glioma patient datasets (Fig. 1L, S2D, S2E).

### ALDH5A1 is a direct target of miR-210 and both show inverse expression correlation in GBM patients

**Fig. 2:**
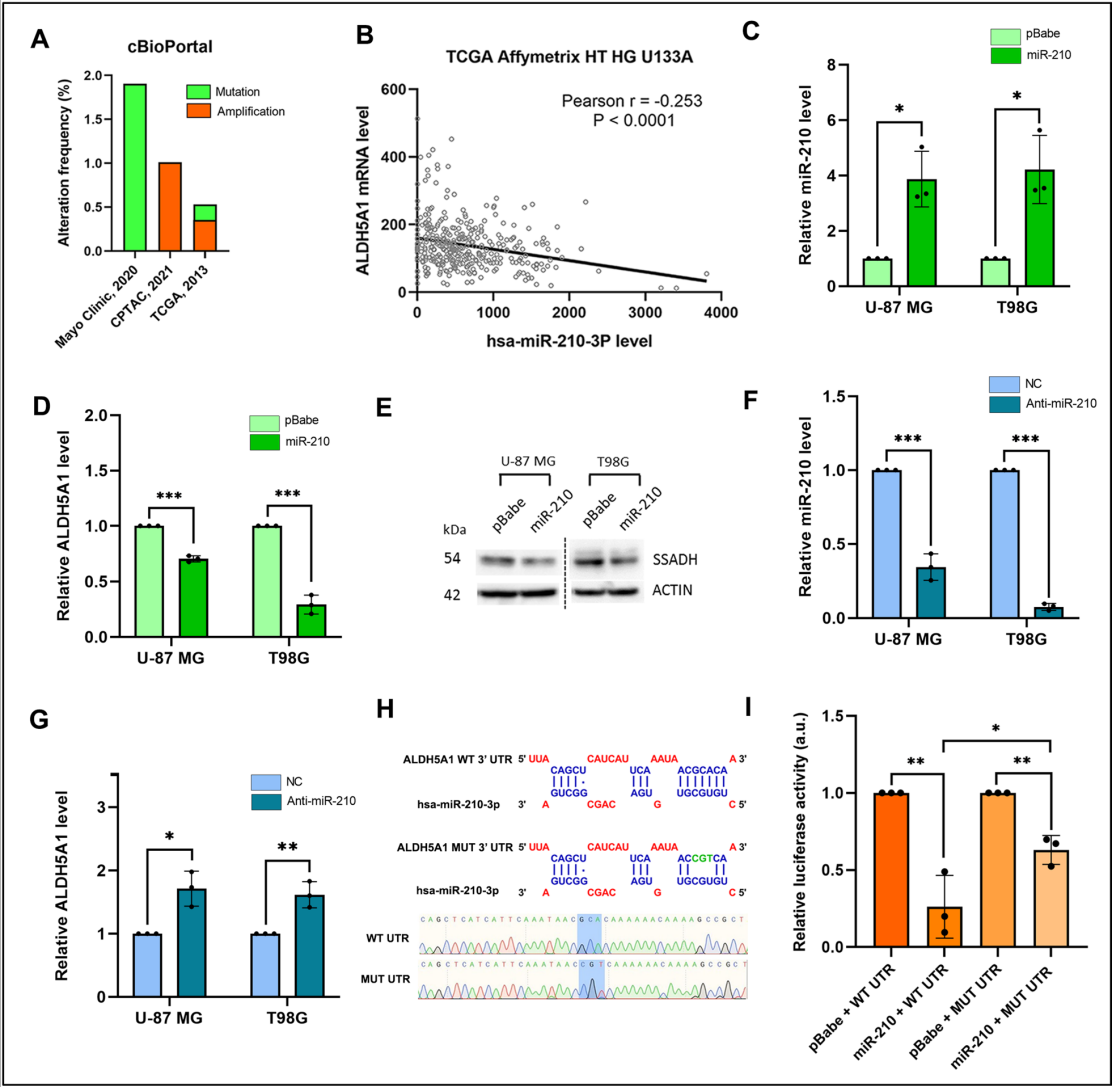
ALDH5A1 is a direct target of miR-210 in GBM: **A** Alteration frequency of ALDH5A1 in GBM patients. **B** Expression levels of ALDH5A1 and miR-210 are inversely correlated in GBM patients. **C** MiR-210 levels in GBM cells upon pre-miR-210-based overexpression. **D** ALDH5A1 mRNA levels in GBM cells upon pre-miR-210-based overexpression. **E** ALDH5A1 (SSADH) protein levels in GBM cells upon pre-miR-210 based overexpression. **F** MiR-210 levels in GBM cells upon anti-miR-based knockdown. **G** ALDH5A1 levels in GBM cells upon anti-miR-210-based knockdown. NC: Negative control. **H** MiR-210 binding site on the 3′ UTR of ALDH5A1 and mutation of binding site by SDM. **I** 3′ UTR-dual luciferase assay showing direct binding of miR-210 to the 3′ UTR of ALDH5A1. (*p > 0.01 and < 0.05, **p < 0.01, ***p < 0.001). Error bars denote ± standard deviation (SD)

We first investigated whether the downregulation of ALDH5A1 is due to genomic alterations such as mutations. We used the cBioPortal tool to study the alteration frequency of this gene in GBM patients. Among the three datasets studied, none had > 2% alteration frequency (Fig. 2A), suggesting the involvement of other possible regulatory mechanisms. Since miRNAs play a critical role as regulators of gene expression, we looked for possible miRNA binding sites in the 3′ UTR of ALDH5A1 transcript using the TargetScanHuman (release 8.0) miRNA target prediction tool. Among the miRNAs predicted to target ALDH5A1 3′ UTR with > 95 context + + score percentile, miR-210-3p was interesting as it has been reported earlier to be involved in mitochondrial dysfunction. We found a canonical binding site of miR-210 in the 3′ UTR of the ALDH5A1 transcript. The 2–8 nucleotides from the 5′ end of miR-210 (seed region) had complete complementarity with the ALDH5A1 3′ UTR region. TargetScanHuman (release 8.0) predicted this interaction to be an 8-mer having a high context + + percentile score of 97 (Figure S3A).

We then investigated the expression correlation of these two in GBM patients. A correlation analysis between miR-210 and ALDH5A1 mRNA levels in 358 TCGA GBM patients (Affymetrix HT HG U133A) revealed a statistically significant negative correlation between them (Pearson *r* = −0.253) (Fig. 2B). This suggested that miR-210 might regulate the expression levels of ALDH5A1 in GBM, and encouraged further validation studies.

To further establish the regulation of ALDH5A1 by miR-210 in GBM, we ectopically modulated expression of miR-210 in the GBM cell lines U-87 MG and T98G. We delivered the precursor sequence of miR-210 cloned in a mammalian expression vector (pBabe-Puro) to the GBM cell lines for overexpressing miR-210. Stem-loop RT-qPCR confirmed that upon transfecting the miR-210 constructs, the levels of mature miR-210 increased in both U-87 MG and T98G cell lines by > 3 folds (Fig. 2C). Upon upregulating miR-210, a significant decrease in ALDH5A1 transcript levels was observed in both cell lines (Fig. 2D). This effect was also verified at protein levels by performing immunoblotting for ALDH5A1 after miR-210 overexpression. We observed a downregulation of ALDH5A1 protein (SSADH, succinic semialdehyde dehydrogenase) in both U-87 MG and T98G cell lines upon miR-210 overexpression (Fig. 2E). Upon delivering anti-miR-210 to U-87 MG and T98G cell lines, we first ensured successful knockdown of endogenous miR-210 levels. By performing stem-loop RT-qPCR we confirmed that in both cell lines, the levels of miR-210 decreased significantly. A 65% downregulation of endogenous miR-210 levels was observed upon delivering anti-miR-210 to U-87 MG cells. On the other hand, in T98G cells this downregulation was > 95% (Fig. 2F). This suggested that anti-miR-210 based knockdown of miR-210 levels in GBM cells was successful. We further measured the effect of this on the transcript levels of ALDH5A1. By performing qRT-PCR, we found that miR-210 knockdown led to an increase of 1.71 and 1.61 folds in the transcript levels of ALDH5A1 in U-87 MG and T98G cells, respectively (Fig. 2G).

We finally verified the interaction between miR-210 and ALDH5A1 by performing a 3′ UTR dual luciferase assay. The 3′ UTR of ALDH5A1 containing miR-210 binding site was cloned downstream of a firefly luciferase gene in the pmiReport luciferase vector. We further mutated the binding site complementary to the miRNA seed region by changing 3 nucleotides (GCA->CGT) with the help of site-directed mutagenesis (SDM) (Fig. 2H). Upon co-transfection of the 3′ UTR construct along with miR-210 in the U-87 MG cell line, we observed a decrease in the luciferase activity (Fig. 2I). When the mutated 3′UTR was co-transfected to U-87 MG cells along with miR-210, a rescue in the luciferase activity was observed (Fig. 2I). This suggested that miR-210 mediated downregulation of ALDH5A1 is through its binding site in the 3′ UTR. Taken together, the above experiments established ALDH5A1 as a direct target of miR-210 in GBM.

### Hypoxia regulates miR-210 and ALDH5A1 expression in GBM

**Fig. 3:**
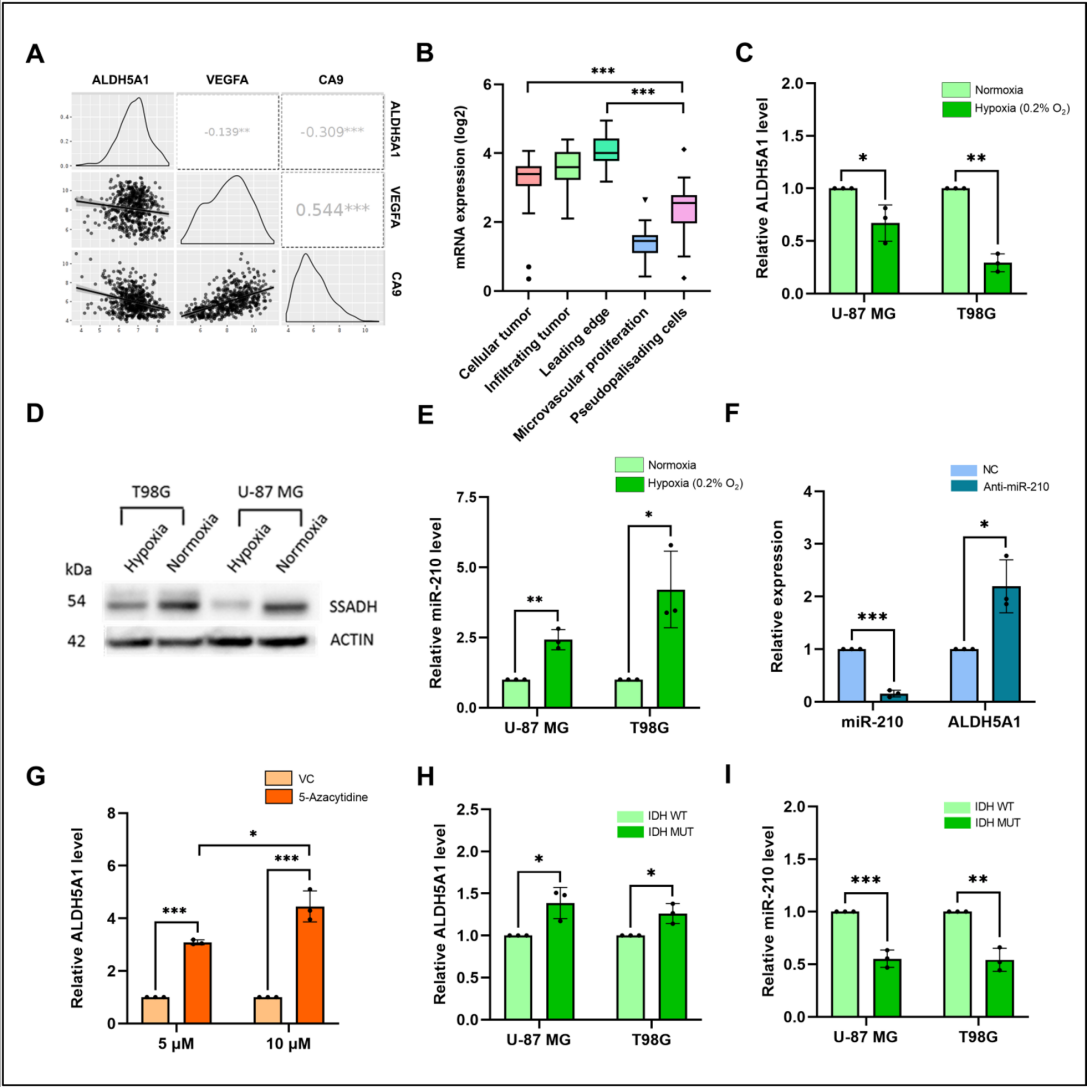
Effect of chronic hypoxia, epigenetic regulation and IDH mutation on ALDH5A1 and miR-210 levels: **A** Correlation of ALDH5A1 expression with hypoxia markers. **B** ALDH5A1 mRNA expression in different tissue sections of GBM. **C** ALDH5A1 is downregulated under hypoxia at the transcript level. **D** ALDH5A1 (SSADH) is downregulated under hypoxia at the protein level. **E** MiR-210 is upregulated under hypoxia in GBM. **F** MiR-210 knockdown under hypoxia increases the transcript levels of ALDH5A1. **G** 5-Azacytidine mediated hypomethylation increases ALDH5A1 transcript levels in a dose-wise manner. **H** ALDH5A1 is upregulated in IDH-mutant GBM cell lines. **I** MiR-210 is downregulated in IDH-mutant GBM cell lines. (*p > 0.01 and < 0.05, **p < 0.01, ***p < 0.001). Error bars denote ± standard deviation (SD)

To study if ALDH5A1 expression is regulated under hypoxic conditions, we first compared the transcript levels of ALDH5A1 with two major markers of hypoxia- VEGFA, and CA9 in GBM patients of the TCGA_GBM dataset using GlioVis web server. A statistically significant negative correlation was observed between ALDH5A1 and VEGFA/CA9 expression (Fig. 3A). Further, we checked its expression in various histological sections of GBM tumor. By exploring the IVY Glioblastoma Project (IVY-GAP) dataset, we observed that ALDH5A1 mRNA expression is significantly downregulated in the hypoxic pseudopalisades of GBM patients, when compared to the leading edge and cellular tumor (Fig. 3B). Finally, we measured the transcript and protein levels of ALDH5A1 under hypoxic conditions. We observed a significant downregulation of ALDH5A1 transcript levels in both U-87MG and T98G cell lines (Fig. 3C). Similar effects were observed at protein levels. A stark decrease in the protein levels of ALDH5A1 was observed in both U-87 MG and T98G cell lines when incubated under hypoxia (Fig. 3D). It is widely known that miR-210 is highly upregulated under hypoxic conditions as the transcription factor HIF1A induces its expression under hypoxia. Since GBM is a solid tumor having a hypoxic core, we verified this by culturing U-87 MG and T98G cells under chronic hypoxia (0.2% O_2_). When compared with normoxic conditions, it was found that miR-210 levels are elevated under hypoxia by > 2 folds in both cell lines (Fig. 3E). We further wanted to check if miR-210 upregulation under hypoxia has any effect on ALDH5A1 expression. For this, we knocked down miR-210 levels under chronic hypoxia and measured ALDH5A1 transcript levels. Interestingly, it was observed that repressing miR-210 under hypoxia using anti-miR-210 led to a significant increase in the transcript levels of ALDH5A1 (Fig. 3F).

### Epigenetic regulation of ALDH5A1 and the effect of IDH mutation

We further wanted to study if ALDH5A1 is regulated by other epigenetic regulatory mechanisms apart from miRNA mediated post-transcriptional silencing. For this, we first investigated the presence of CpG islands in the promoter region of ALDH5A1. Using the Database of CpG islands and Analytical Tool (DBCAT), we identified a dense CpG island in the promoter region of ALDH5A1 (Figure S3B). In order to understand whether the CpG Island present in the promoter of ALDH5A1 is methylated, we treated the T98G GBM cells using increasing dosage of the global hypomethylation agent 5-Azacytidine and its vehicle control (DMSO). Interestingly, we found that with increasing dose of 5-Azacytidine, the transcript levels of ALDH5A1 increased (Fig. 3G). This suggested that ALDH5A1 downregulation in GBM might be attributed to epigenetic regulation of its expression by promoter methylation.

Although the 2021 WHO classification of CNS tumors has discarded the term ‘IDH mutant glioblastoma’, mutation in this gene has prognostic significance in glioma patients. Patients harboring an IDH1 R132H mutation have been observed to have a better prognosis than their wild type counterpart. The IDH1 (isocitrate dehydrogenase 1) is a crucial enzyme of the TCA cycle that converts isocitrate to α-ketoglutarate (α-KG), releasing NADPH in the process. The mutant IDH1 however converts α-KG into the oncometabolite D-2-hydroxyglutarate. Studies have suggested that R132H mutation promotes the uptake of lactate and glutamate instead of glucose to facilitate the TCA cycle. Since ALDH5A1 codes for the mitochondrial enzyme succinic semialdehyde dehydrogenase (SSADH) which converts succinate semialdehyde to succinate, feeding the TCA cycle, we were interested to check its expression in IDH mutant cells. For this, we overexpressed a wild type and R132H mutant clone of IDH1 into GBM cells and measured ALDH5A1 and miR-210 transcript levels. We found that ALDH5A1 is significantly upregulated in IDH mutant group of cells in both cell lines (Fig. 3H). Interestingly, we also observed that miR-210 is significantly downregulated in IDH mutant group of cells (Fig. 3I). An analysis of ALDH5A1 and MIR210 RNA in GBM patients of Bao dataset (data downloaded from GlioVis; IDH1 WT (*n* = 70); IDH Mut (*n* = 26) revealed ALDH5A1 to be upregulated (Figure S3C) and MIR210 to be downregulated (Figure S3D) in IDH1 mutant patients as compared to the wild-type.

### Protein-protein Interaction and functional enrichment of ALDH5A1

**Fig. 4:**
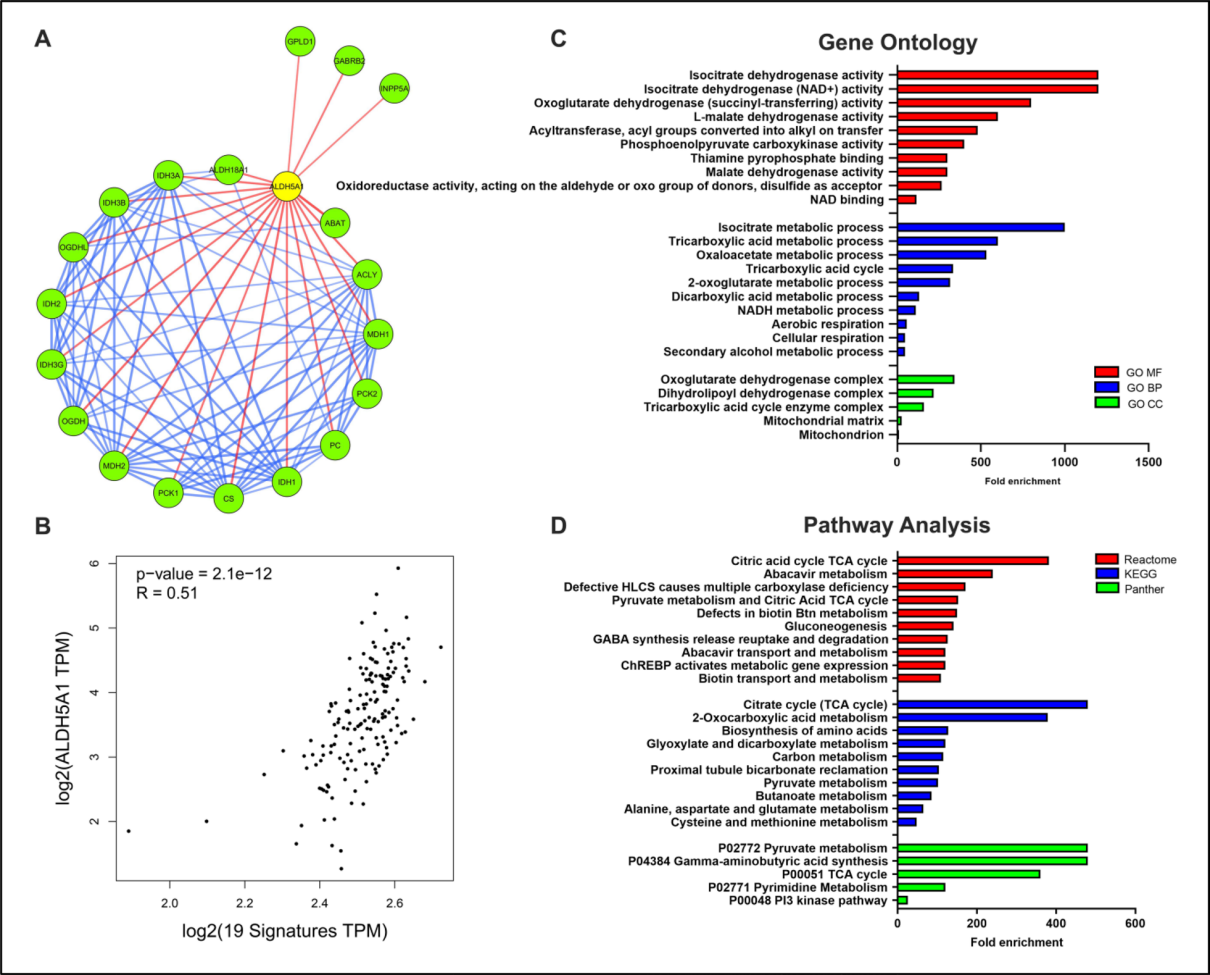
***In silico ***Protein-protein interaction and functional enrichment of ALDH5A1: **A** PPI network of ALDH5A1 and its interacting proteins (STRING database, confidence cutoff = high). **B** Correlation of ALDH5A1 transcript expression with its interacting partners in GBM patients (GEPIA2 database). **C** Gene ontology and **D** Pathway analysis of ALDH5A1 and interacting proteins. Data downloaded from ShinyGO web server

In order to understand the possible biological role of ALDH5A1 in GBM, we performed an *in silico* protein-protein interaction (PPI) study of ALDH5A1 using the STRING database. When a high confidence cutoff was applied, a total of 19 interacting proteins were identified that are mostly involved in cellular metabolism (Fig. 4A). Interestingly, several proteins of the isocitrate dehydrogenase (IDH) family such as IDH1, IDH2, IDH3A, IDH3B, and IDH3G, were found to be interacting with ALDH5A1. We further investigated how these interacting partners correlate clinically based on their expression. A correlation analysis in the GEPIA2 web portal revealed that these 19 genes had significant positive correlation with ALDH5A1 at the transcript level in GBM patients (Fig. 4B). To get a better look at the biological processes and pathways maintained by ALDH5A1 and its interacting proteins, we performed a gene ontology (GO) and pathway enrichment analysis of the above set of 20 proteins using the ShinyGO web-tool. Interestingly, we found that the most enriched molecular function (GO MF) was isocitrate dehydrogenase activity. Among the other enriched MFs, various dehydrogenase activities such as oxoglutarate dehydrogenase (succinyl-transferring) and L-malate dehydrogenase were observed (Fig. 4C). Isocitrate metabolic process, tricarboxylic acid metabolic process, and oxaloacetate metabolic process were among the top enriched biological processes (GO BP) (Fig. 4C). Among the top enriched cellular components (GO CC), oxoglutarate dehydrogenase complex, tricarboxylic acid cycle enzyme complex, and mitochondrial matrix were notable (Fig. 4C). Reactome, KEGG, and Panther pathway analysis revealed important pathways such as citrate cycle (TCA cycle), pyruvate metabolism, carbon metabolism and GABA synthesis release uptake and degradation, among others. Interestingly, TCA cycle and pyruvate metabolism were found to be common among all the pathways (Fig. 4D). Thus, we were interested in studying the possible role of ALDH5A1 in GBM metabolism. MiR-210 has been identified previously as a negative regulator of mitochondrial metabolism in other cancers. So, we were further interested to study the role of ALDH5A1/miR-210 axis in the context of GBM metabolism.

### ALDH5A1 inhibits while miR-210 promotes aerobic glycolysis of GBM cells

Previous reports have suggested that miR-210 inhibits mitochondrial respiration and might be indirectly involved in promoting the rate of glycolysis. Since ALDH5A1 is a mitochondrial enzyme and it was found to be a direct target of miR-210, we were further interested to investigate the role of miR-210/ALDH5A1 in aerobic glycolysis of GBM cells. We conducted a series of metabolic assays to investigate the role of ALDH5A1/miR-210 axis in GBM metabolism. Before proceeding with functional assays, ALDH5A1 overexpression was confirmed by immunoblotting where strong induction of ALDH5A1 protein was observed in the overexpression group as compared to the empty vector control (Figure S3E).

**Fig. 5:**
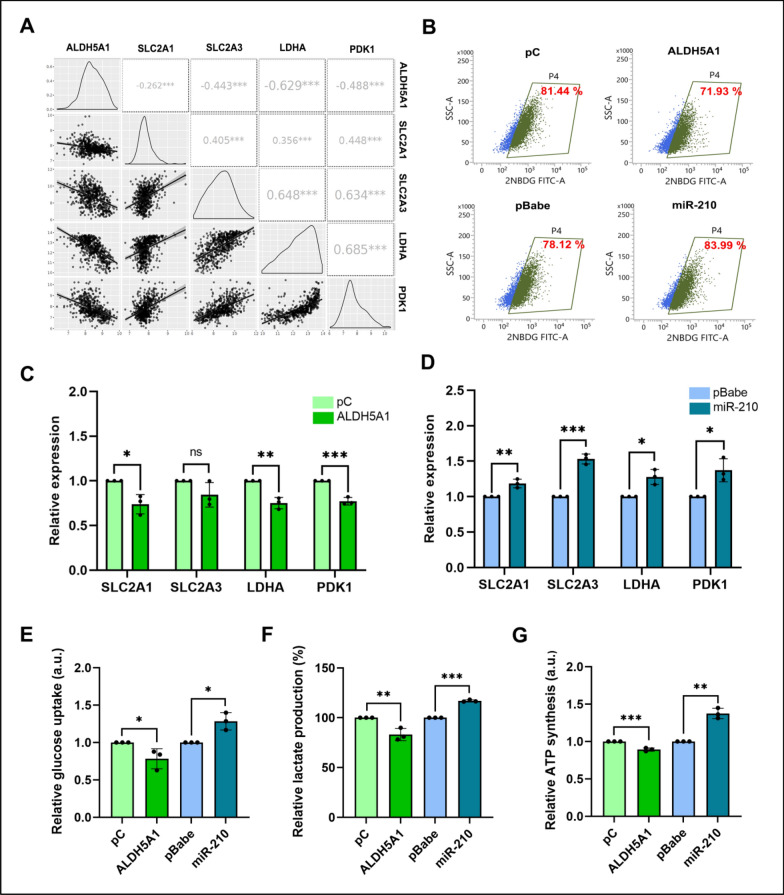
Effect of ALDH5A1 and miR-210 overexpression on glycolysis: **A** Expression correlation analysis of ALDH5A1 with glucose transporters and glycolysis markers in GBM patients of Rembrandt dataset. Data was downloaded from GlioVis web server. **B** Flow-cytometric analysis of glucose uptake in GBM cells upon ALDH5A1 and miR-210 overexpression. **C** Effect of ALDH5A1 and **D** miR-210 overexpression on the transcript levels of glucose transporters and glycolysis markers. **E** Relative glucose uptake of GBM cells upon ALDH5A1 and miR-210 overexpression. **F** Relative lactate production of GBM cells upon ALDH5A1 and miR-210 overexpression. **G** Effect of ALDH5A1 and miR-210 overexpression on total ATP synthesis of GBM cells. (*p > 0.01 and < 0.05, **p < 0.01, ***p < 0.001). Error bars denote ± standard deviation (SD)

### Glucose uptake

Glucose is the primary carbon source used for generating ATP, the energy currency of the cell. Cells that undergo rapid glycolytic flux to maintain high proliferation rate import more glucose through the glucose transporters. To measure glucose uptake, we first studied the expression correlation of two major glucose transporters in GBM- solute carrier family 2 member 1 (SLC2A1) and solute carrier family 2 member 3 (SLC2A3) (previously known as GLUT1 and GLUT3) with ALDH5A1 levels. By analyzing the transcriptomic data from the Rembrandt dataset, we found a significant negative correlation of ALDH5A1 mRNA levels with both SLC2A1 and SLC2A3, while the latter two shared a positive correlation between them (Fig. 5A). We further overexpressed both ALDH5A1 and miR-210 in the U-87 MG GBM cell line to study their effects on cellular glucose uptake. First, we performed a flow-cytometry based 2-[N-(7-nitrobenz-2-oxa-1,3-diazol-4-yl) amino]-2-deoxy-D-glucose (2-NBDG) uptake study. 2-NBDG functions as a fluorescent analog of glucose, employed for tracking glucose absorption in viable cells in real-time. Higher uptake of 2-NBDG in no-glucose media was observed more in miR-210 overexpressing group of cells (a 5.87% increase), while cells having ALDH5A1 overexpression showed a 9.51% decrease in the import of the compound as compared to their respective controls (Fig. 5B). Next, we measured the transcript levels of SLC2A1 and SLC2A3 upon ALDH5A1 and miR-210 overexpression. It was found that upon ALDH5A1 overexpression, the levels of SLC2A1 transcript got significantly repressed (Fig. 5C), while miR-210 overexpression led to an increase in the transcript levels of both SLC2A1 and SLC2A3 (Fig. 5D). Moreover, through a colorimetric biochemical assay that employs an improved o-toluidine method, we measured glucose uptake of cells overexpressed with ALDH5A1 and miR-210. It was found that miR-210 overexpression led to higher glucose uptake into U-87 MG cells, while ALDH5A1 overexpression resulted in opposite observation (Fig. 5E). This experiment finally suggested that ALDH5A1 overexpression reduces the uptake of glucose in cells while miR-210 overexpression promotes it.

### Lactate production

Increased lactate production is a direct indicator of highly glycolytic cells. For cancer cells which often have high glycolytic index, the pyruvate molecule is actively converted to lactate with the help of the lactate dehydrogenase (LDH) enzyme. On the other hand, pyruvate dehydrogenase (PDH), the enzyme responsible for converting pyruvate to acetyl-coA and thereby feeding the Krebs cycle, is inactivated by pyruvate dehydrogenase kinase (PDK) mediated phosphorylation. This helps in maintaining high glycolytic levels of actively proliferating cancer cells. We therefore focused on LDHA and PDK1; two very important enzymes that decide the fate of pyruvate and help decide whether cells will undergo glycolysis or OXPHOS. We first studied the expression correlation of LDHA and PDK1 with ALDH5A1 levels in GBM patients. By analyzing the transcriptomic data from the Rembrandt dataset, we found a significant negative correlation of ALDH5A1 mRNA levels with both LDHA and PDK1, while the latter two shared a positive correlation between them (Fig. 5A). Next, we overexpressed both ALDH5A1 and miR-210 in the U-87 MG GBM cell line to study their effects on the transcript levels of LDHA and PDK1. It was found that upon ALDH5A1 overexpression, the transcript levels of both LDHA and PDK1 got significantly repressed (Fig. 5C), while miR-210 overexpression led to an increase in the transcript levels of both LDHA and PDK1 (Fig. 5D). Cells export the generated lactate into extracellular space with the help of monocarboxylate transporters (MCTs) to avoid toxicity. This results in an increase in the extracellular lactate. We therefore measured lactate in the supernatant of miR-210 and ALDH5A1 overexpressed U-87 MG GBM cells. Interestingly, it was found that ALDH5A1 overexpression decreased the extracellular lactate formation while miR-210 overexpression increased it (Fig. 5F).

### ATP synthesis

Although the total ATP generated from the TCA cycle far exceeds than that of glycolysis, the oxidative phosphorylation process is rather slow, and aggressively proliferating cancer cells depend more on glycolysis for rapid ATP turnover. We therefore measured the total ATP production of GBM cells upon miR-210 and ALDH5A1 overexpression in a short time frame (24 h). It was observed that ALDH5A1 overexpression led to a marginal decrease in total ATP formation of both U-87 MG and T98G cell lines. On the other hand, miR-210 overexpression resulted in increased ATP formation of GBM cells (Fig. 5G).

### Extracellular acidification rate (ECAR)

Cellular glucose is transformed into pyruvate through glycolysis, and this pyruvate can then be further converted into lactate within the cell’s cytoplasm, or into carbon dioxide and water within the mitochondria. This process of glucose conversion, first to pyruvate and then possibly to lactate, leads to the release of protons, which are pushed out into the extracellular space. This proton release contributes to the increased acidity of the environment surrounding the cell. The XF instrument gauges this process by directly assessing the rate of acidification and presents it as ECAR (mpH/min), which is considered as the gold standard for assessing glycolytic rate of a living cell.

**Fig. 6:**
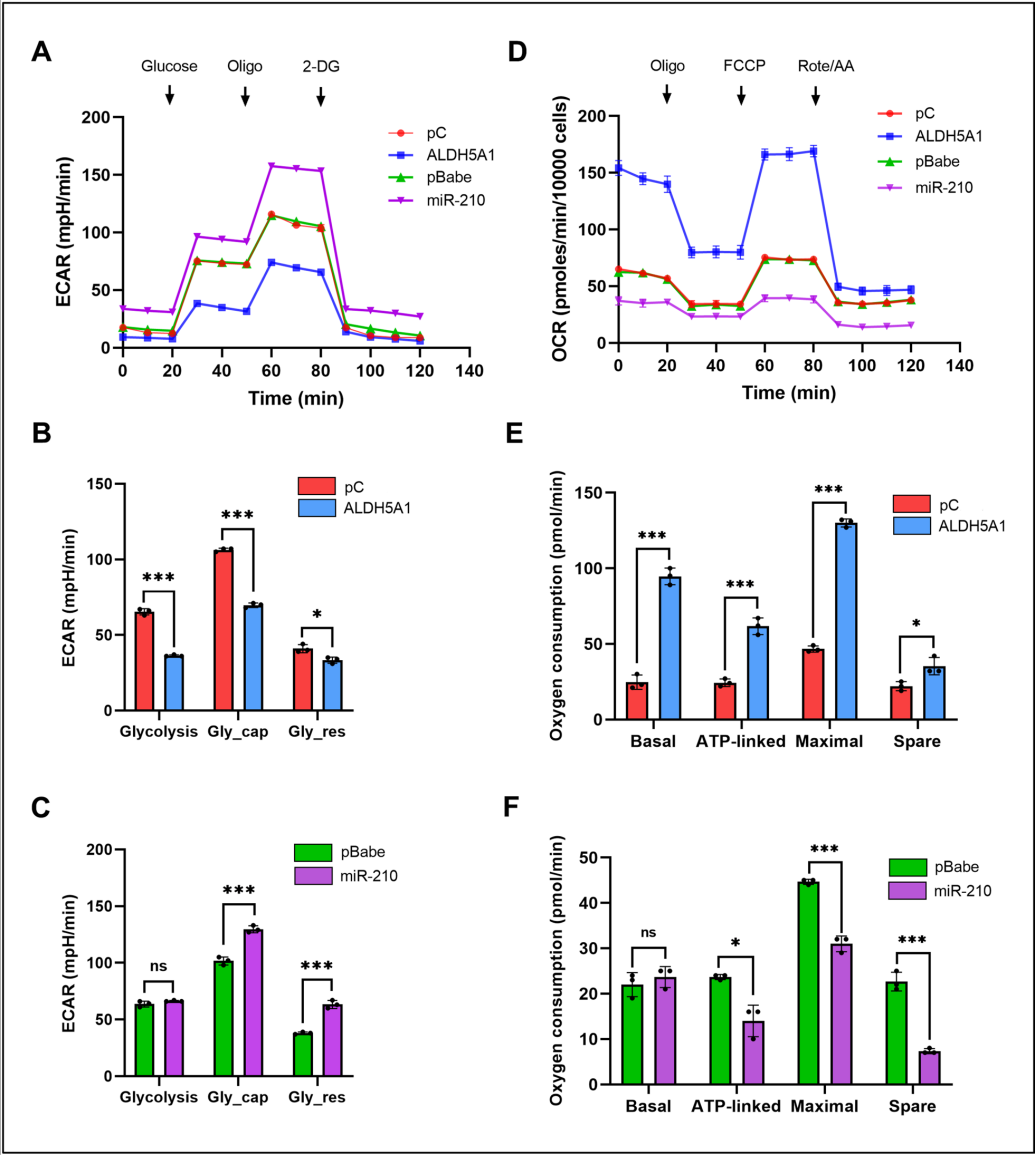
Effect of ALDH5A1 and miR-210 overexpression on ECAR and OCR of GBM cells: **A** ECAR profile of U-87 MG GBM cells transfected with ALDH5A1 and miR-210 along with empty vector controls. **B** Measurement of glycolysis, glycolytic capacity and glycolytic reserve of control and ALDH5A1 overexpressed cells. **C** Measurement of glycolysis, glycolytic capacity and glycolytic reserve of control and miR-210 overexpressed cells. **D** OCR profile of U-87 MG GBM cells transfected with ALDH5A1 and miR-210 along with empty vector controls. **E** Measurement of basal, ATP-linked, maximal and spare respiration of control and ALDH5A1 overexpressed cells. **F** Measurement of basal, ATP-linked, maximal and spare respiration of control and miR-210 overexpressed cells. (*p > 0.01 and < 0.05, **p < 0.01, ***p < 0.001). Error bars denote ± standard deviation (SD)

We measured the ECAR of GBM cells transfected with ALDH5A1 and miR-210, along with their respective controls. Interestingly, we found that the maximum ECAR was achieved in cells transfected with miR-210, while ALDH5A1 transfected cells exhibited minimum ECAR. On the other hand, the empty vector controls displayed similar ECAR profiles (Fig. 6A). Next we calculated the basal glycolysis (glycolysis), glycolytic capacity (Gly_cap) and glycolytic reserve (Gly_res), by analyzing the raw data. When ALDH5A1 was overexpressed, in U-87 MG GBM cells, we observed a significant reduction in the basal rate of glycolysis, glycolytic capacity as well as glycolytic reserve (Fig. 6B). On the other hand, overexpression of miR-210 in U-87 MG cells resulted in an increased glycolytic capacity and glycolytic reserve. However, we did not observe a significant change in the basal level of glycolysis (Fig. 6C). Taken together, the above experiment showed that ALDH5A1 reprograms GBM cells to undergo less glycolysis, while miR-210 can support higher glycolytic rates.

### Oxygen consumption rate (OCR)

The OCR is a crucial parameter in cellular metabolism that reflects the rate at which cells utilize oxygen during various biochemical processes. OCR is primarily measured to gain insights into cellular respiration, a fundamental energy-producing mechanism in which cells use oxygen to generate ATP, the cell’s primary energy currency. By quantifying OCR, we can evaluate the efficiency of the OXPHOS and metabolic pathways within the mitochondria, offering valuable information about the cell’s overall energy status, metabolic health, and responses to different stimuli or conditions. Our previous findings indicated that ALDH5A1 reprograms cellular metabolism by reducing the glycolysis of GBM cells while miR-210 promoted it. Since ALDH5A1 codes for the mitochondrial SSADH which indirectly feeds the TCA cycle by providing succinate through the GABA-shunt, we were interested in studying the roles of ALDH5A1/miR-210 in mitochondrial respiration.

We measured the OCR of GBM cells transfected with ALDH5A1 and miR-210, along with their respective controls. Interestingly, we found that the maximum OCR was achieved in cells transfected with ALDH5A1, while miR-210 transfected cells exhibited minimum OCR. On the other hand, the empty vector controls displayed similar OCR profiles (Fig. 6D). Next, we calculated the OCR linked with basal respiration, ATP-linked respiration, Maximal respiration and the spare respiratory capacity, by analyzing the raw data. When ALDH5A1 was overexpressed, in U-87 MG GBM cells, we observed a significant increase in the basal, ATP-linked, maximal as well as spare respiratory capacity (Fig. 6E). On the other hand, overexpression of miR-210 in U-87 MG cells resulted in a decreased ATP-linked, maximum, and spare respiration. However, we did not observe a significant change in the basal level of respiration (Fig. 6F). Taken together, the above experiment showed that ALDH5A1 promotes the mitochondrial respiration of GBM cells, while miR-210 inhibits it.

### ALDH5A1 inhibits cellular proliferation of GBM

**Fig. 7:**
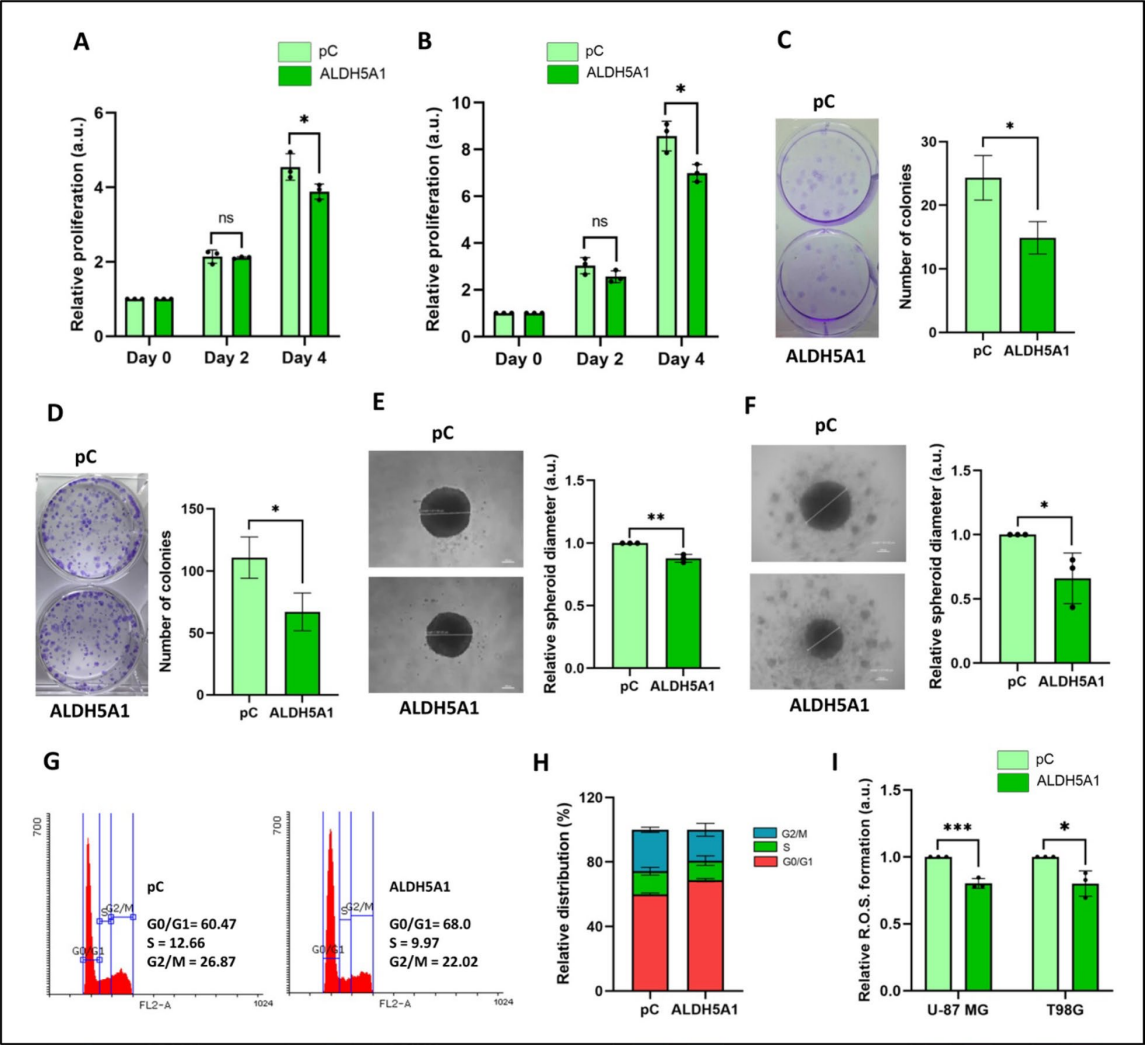
ALDH5A1 overexpression inhibits proliferation, spheroid formation and ROS levels in GBM: **A** ALDH5A1 inhibits cellular proliferation of U-87 MG cells. **B** ALDH5A1 inhibits cellular proliferation of T98G cells. Representative image of colony formation assay and quantification of colony numbers: **C** U-87 MG and **D** T98G cells. ALDH5A1 inhibits 3D spheroid formation and quantification of relative spheroid diameter: **E** U-87 MG and **F** T98G cells. **G** ALDH5A1 promotes G0/G1 cell cycle arrest of T98G cells. **H** Quantification of distribution of cells in various phases of the cell cycle upon ALDH5A1 overexpression. **I** ALDH5A1 inhibits ROS formation in GBM cells. (*p > 0.01 and < 0.05, **p < 0.01, ***p < 0.001). Error bars denote ± standard deviation (SD)

As the functional role of miR-210 in GBM has been well reported [8–12], we were more interested in studying the functional role of ALDH5A1 in GBM. Since cellular proliferation is directly linked with the metabolism of cancer cells, we mainly studied the role of ALDH5A1 in regulating the proliferative capaity of GBM cells. We overexpressed ALDH5A1 in U-87 MG and T98G GBM cells and performed MTT-based cellular proliferation assay. It was observed that ALDH5A1 overexpression significantly inhibited the cell proliferation of both U-87 MG (Fig. 7A) and T98G cells (Fig. 7B) at day 4. The colony formation assay provides insights into the ability of cancer cells to survive, divide, and generate new colonies, reflecting their clonogenic potential and capacity for uncontrolled replication. We measured the colony forming ability of GBM cells after overexpressing ALDH5A1. It was observed that ALDH5A1 overexpression inhibited the clonogenic potential of both U-87 MG (Fig. 7C) and T98G (Fig. 7D) GBM cells. This finding supported our previous observation that ALDH5A1 overexpression reduced the glycolytic potential of GBM cells while promoting mitochondrial respiration. Reprogramming the primary mode of metabolism from glycolysis to OXPHOS might be responsible for the anti-proliferative effect of ALDH5A1.

###  ALDH5A1 decreases spheroid formation of GBM cells

The 3D spheroid formation assay has emerged as a dynamic approach for assessing cancer cell proliferation within a more physiologically relevant context. When the cells are grown in the absence of a substratum in an anchorage independent manner, over time, they aggregate and self-assemble into compact spheroid structures, better representing the in vivo conditions compared to traditional two-dimensional monolayer cultures. The ability of cancer cells to form and grow as 3D spheroids is a measure of their proliferation and aggressiveness. We overexpressed ALDH5A1 in GBM cells and observed that ALDH5A1 overexpression reduced the spheroid forming ability of both U-87 MG (Fig. 7E) and T98G (Fig. 7F) cells. However, the decrease in spheroid diameter was more prominent in T98G cells as compared to U-87 MG.

### ALDH5A1 induces G0/G1 cell cycle arrest in GBM cells

Cell cycle analysis serves as a crucial method for quantifying cancer cell proliferation by examining the distribution of cells across different phases of the cell cycle. Accelerated proliferation in cancer cells often leads to alterations in the cell cycle distribution, such as a higher percentage of cells in S or M phases. We studied the effect of ALDH5A1 overexpression on the cell cycle of T98G GBM cells. It was observed that as compared to control (pC), approximately 8% more cells were arrested in the G0/G1 phase in ALDH5A1 transfected T98G GBM cells (Fig. 7G and H). This increase in cells in the G0/G1 phase might be due to anti-proliferative potential of ALDH5A1 in GBM.

### ALDH5A1 decreases reactive oxygen species (ROS) formation in GBM

Reactive oxygen species (ROS) derived from the partial reduction of oxygen molecule gives rise to free radicals that stimulate a broad spectrum of activity in cancer cells. ROS might lead to oncogene activation, and increased metabolism of cancer cells. Increased ROS initially promotes tumor progression, however after its excessive accumulation leads to cytotoxicity. Thus we were interested to understand the role of ALDH5A1 in the formation and management of cellular ROS. By using a spectroscopic method, we measured the ROS levels of GBM cells upon ALDH5A1 overexpression. Interestingly, we found a significant decrease in the ROS levels of cells having high levels of ALDH5A1. A spectroscopic analysis revealed that ALDH5A1 overexpression reduces ROS formation in both U-87 MG and T98G (Fig. 7I) cells. This might be due to the fact that ALDH5A1 metabolizes the toxic aldehydes in cells that contribute to higher ROS levels. Thus, ALDH5A1 acts as an anti-oxidant molecule in GBM.

**Fig. 8:**
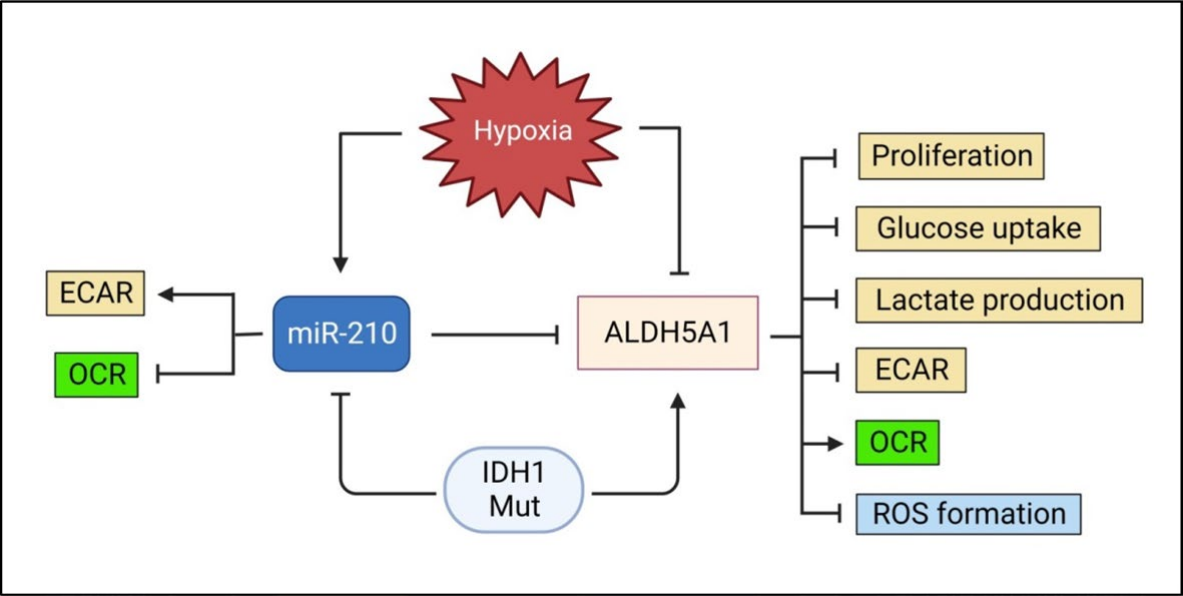
Schematic diagram summarizing the role of miR-210/ALDH5A1 axis in GBM metabolism

## Discussion

Altered metabolism has emerged as a hallmark of cancer due to its profound impact on tumor development and progression. Unlike normal cells, cancer cells exhibit distinct metabolic reprogramming, characterized by increased glucose uptake, enhanced glycolysis even in the presence of oxygen (the Warburg effect), and altered utilization of nutrients to support rapid proliferation. These metabolic adaptations provide cancer cells with a competitive advantage by supplying the energy and building blocks necessary for their unchecked growth and survival. Previous studies have identified that GBM maintains a high glycolytic phenotype for meeting the exorbitant energy demands of its rapidly proliferating cells [13, 14]. Some studies have suggested that the presence of a hypoxic core and the resultant HIF-signaling helps to maintain a high glycolytic ratio in GBM [15, 16]. Identification of novel players involved in metabolic rewiring of GBM tumors will advance our understanding of tumor progression and open novel avenues of therapeutic treatment.

To this end, we identified ALDH5A1 as a predicted target of the hypoxia regulated miR-210 which might be involved in GBM metabolism. Aldehyde dehydrogenases (ALDHs) comprise a group of enzymes responsible for preserving cellular balance through the processing of both naturally occurring and externally introduced reactive substances. As of now, the human ALDH superfamily is recognized to contain 19 potentially functional genes, organized into 11 primary families and 4 subfamilies. Different families carry out distinct functions and are involved in various disorders. For example, various genes of the ALDH1 family have been identified to play key roles in stemness and drug resistance in cancers. On the other hand, the ALDH2 family is involved in alcohol sensitivity, while ALDH7 is involved in hyperosmotic stress [17]. ALDH5A1 is a member of ALDH5 family that codes for a mitochondrial enzyme known as SSADH, involved in the metabolism of the inhibitory neurotransmitter GABA. A deficiency in SSADH levels leads to improper GABA metabolism and as a result might cause severe cognitive impairment. We found that ALDH5A1 is highly expressed in the human brain; however, its levels are significantly downregulated in GBM. Also, as a by-product of GABA metabolism, ALDH5A1 feeds the TCA cycle with succinate, thereby promoting oxidative phosphorylation. We therefore hypothesized that miR-210 might promote glycolysis of GBM by regulating ALDH5A1 levels. MiR-210 is an oncogenic miRNA in GBM whose expression is induced under hypoxia and has an overall elevated expression in GBM patients as compared to the normal brain. To the best of our knowledge, till date, no study has explored the role of miR-210 in GBM metabolism. However, some earlier studies have commented on its role in inhibiting mitochondrial respiration of other cancers. In 2010, Chen et al. had shown that miR-210 mediated targeting of COX10 (cytochrome c oxidase assembly protein) and ISCU (iron-sulfur cluster scaffold homolog) impairs mitochondrial function and promotes glycolysis of colon cancer cells [18]. Puissegur et al. have also shown that miR-210 negatively regulates mitochondrial metabolism of lung cancer cells by targeting SDHD (subunit D of succinate dehydrogenase complex), a component of the ETC [19]. Based on the existing evidences in other cancers, we were interested in exploring the ALDH5A1/miR-210 axis in GBM, possibly uncovering its role in altered metabolism.

We established ALDH5A1 as a direct target of miR-210 in GBM using techniques like qRT-PCR, western blotting, 3’ UTR dual luciferase assay and SDM. Our findings revealed that ALDH5A1 is heavily downregulated under hypoxia. We confirmed that the hypoxic suppression of ALDH5A1 transcript and protein was driven by miR-210 as knocking down miR-210 under hypoxia increased ALDH5A1 levels. In order to study the role of miR-210/ALDH5A1 in aerobic glycolysis and mitochondrial metabolism, we overexpressed both in GBM cells. Our findings reveal for the first time that ALDH5A1 overexpression negatively regulates glycolysis of GBM cells as evidenced by reduced glucose uptake and lactate production. On the other hand, miR-210 overexpression promoted glucose uptake of GBM cells and enhanced lactate production. Measurement of ECAR revealed that ALDH5A1 significantly decreased the glycolysis of GBM cells, while miR-210 significantly increased their glycolytic capacity and reserve. This essentially confirms that under conditions that require rapid supply of energy, miR-210 can promote glycolysis of GBM cells to meet such demands. Further investigation uncovered that ALDH5A1 significantly increases the oxygen consumption of GBM cells, thereby promoting the OXPHOS process. On the other hand, miR-210 decreased oxygen consumption in the ATP-linked steps of OXPHOS, and also reduced the spare and maximal capacity of mitochondrial respiration. Notably, Voloboueva et al. demonstrated in 2017 that inhibition of miR-210 increases the mitochondrial membrane potential of neural stem cells [20]. Our findings corroborate with earlier studies demonstrating miR-210’s role in negatively regulating mitochondrial respiration in other cancers [18, 19]. Interestingly, we also found that ALDH5A1 expression increased when an R132H mutated clone of IDH1 was transfected into GBM cells as compared to the wild-type variant. On the other hand, miR-210 levels decreased in the IDH-mutant group of cells. This result holds clinical significance as IDH mutation is associated with favorable prognosis of glioma patients.

As the functional role of miR-210 in GBM is well explored and it is established as an oncomiR, we were interested in studying the role of ALDH5A1 in GBM progression. Since it was found to be inhibiting the glycolysis of GBM cells, we primarily focused on its role in regulating cellular proliferation. Indeed, we observed that ALDH5A1 overexpression significantly reduced the proliferation, clonogenic potential and 3D-spheroid forming ability of both U-87 MG and T98G GBM cells. However, this inhibitory effect on cellular proliferation was more pronounced in T98G cells in comparison to U-87 MG. The decrease in proliferation might be due to the reduced glycolysis of GBM upon ALDH5A1 overexpression. Our observation also revealed that ALDH5A1 overexpression reduced ROS formation in GBM cells. This might be due to its ability to metabolize toxic aldehydes that give rise to ROS in cells. A recent study by Menduti et al. in 2020 also reported that ALDH5A1 has possible anti-oxidant roles in GBM cells [21]. In 2023, Piperi et al. have shown that ALDH5A1 is upregulated in GBM and its expression increases in a grade-wise manner. They also show that knocking down ALDH5A1 inhibits the migratory potential of GOS-3 and SJ-GBM2 cell lines, however, no significant change was observed in T98G cells [22]. However, this result contradicts with *Tayrac et al.* where they have shown that ALDH5A1 is downregulated in high grade glioma (grade IV) as compared to grade III. Through univariate Cox analysis, they also identified that ALDH5A1 was one of the 5 protective genes in glioma that is involved the GO biological process ‘*nervous system development’* [23]. Moreover, high ALDH5A1 levels have been associated with better OS of ovarian cancer patients [24]. Although there are some controversial observations regarding ALDH5A1 expression in glioma, our analysis of multiple patient datasets suggest that ALDH5A1 is significantly downregulated in GBM compared to the normal brain, and its low levels are associated with poor prognosis. Moreover, we have shown that ALDH5A1 is downregulated in Indian patients at the transcript level and also in GBM cell lines when compared to normal brain RNA. Our findings suggest that ALDH5A1 overexpression significantly reduces glycolysis in GBM cells which might be one of the reasons of reduced proliferation observed.

As discussed earlier, studies have shown that GBM tumors usually maintain a high glycolytic phenotype, and several mechanisms might be involved in this process, miRNA-mediated regulation being one of them. Our study discovers a novel angle in this context, as we show that miR-210 mediated targeting of the mitochondrial ALDH5A1 reprograms cellular metabolism. This perhaps strengthens the earlier observations suggesting that miR-210 impairs mitochondrial respiration by targeting key OXPHOS genes like ISCU, COX10, and SDHD. The low levels of ALDH5A1 in GBM might be partially due to its targeting by miR-210, which is highly expressed in this cancer. As ALDH5A1 feeds the TCA cycle by metabolizing GABA and supplying succinate, its downregulation by miR-210 might be one of the reasons that shift the energy balance of GBM cells from OXPHOS to glycolysis. In SSADH deficiency, severe cognitive impairment and symptoms like seizure are observed, which is not uncommon in GBM. Thus, miR-210 mediated downregulation of ALDH5A1 in GBM can be one of the factors responsible for these symptoms. Overall, our study shows for the first time that miR-210 mediated targeting of the mitochondrial ALDH5A1 reprograms the metabolism of GBM cells and also uncovers the tumor suppressive role of ALDH5A1 in GBM (Fig. 8).

## Conclusions

In summary, our study unveils a novel ALDH5A1/miR-210 axis that plays a crucial role in metabolic rewiring of GBM. By establishing ALDH5A1 as a direct target of the oncogenic miR-210, and studying their roles in cellular metabolism, we demonstrate that downregulation of ALDH5A1 by miR-210 likely contributes to the observed shift towards glycolysis and impaired mitochondrial respiration in GBM. Furthermore, we show that ALDH5A1 is downregulated in GBM patients, acts as a tumor suppressor in cell lines, and its high expression correlates with improved patient survival, further strengthening its clinical significance. These findings highlight the potential of targeting the ALDH5A1/miR-210 axis for therapeutic intervention in GBM. Upregulating ALDH5A1 or inhibiting miR-210 could offer promising strategies to combat GBM by promoting mitochondrial respiration and reducing glycolysis, potentially improving patient outcomes.

### Supplementary Information

Supplementary Material 1.

Supplementary Material 2.

## Data Availability

No datasets were generated or analysed during the current study.
