# Supplementary material for: ALDH5A1/miR-210 axis plays a key role in reprogramming cellular metabolism and has a significant correlation with glioblastoma patient survival
Source: Cancer Cell Int. 2024 Jul 22;24:259. doi: 10.1186/s12935-024-03432-z (PMC11265472; doi:10.1186/s12935-024-03432-z)
Supplement: Supplementary file 1 — Supplementary Material 1. [file 12935_2024_3432_MOESM1_ESM.pdf]

ALDH5A1/miR-210 axis plays a key role in reprogramming cellular metabolism and has a significant correlation with glioblastoma patient survival

Indranil Mondal [1], Neelam Gupta [2, 3], Vikas Sharma [4], Chitra Sarkar [5], Durga Prasad Mishra [2, 3], and Ritu Kulshreshtha [1*]

*Corresponding author: Dr. Ritu Kulshreshtha
Department of Biochemical Engineering and Biotechnology
Indian Institute of Technology Delhi
Email: ritu@dbeb.iitd.ac.in , drritukulshreshtha@gmail.com

APPENDIX I

List of qPCR primers

Gene name	Forward Primer (5'-3')	Reverse Primer (5'-3')
ALDH5A1	GGCACCAGTTATCAAGTTCG	TTCGTTGACGCCAACCATG
SLC2A1	CTTCACTGTCGTGTCGCTG	GGCCACGATGCTCAGATAG
SLC2A3	GTGGAGAAAACTTGCTGCTGAG	CTGGGGTGACCTTCTGTGTCC
LDHA	GGATGAGCTTGCCCTTGTTGA	GACCAGCTTGGAGTTCGCAGTTA
PDK1	CATGTCACGCTGGGTAATGAGG	CTCAACACGAGGTCTTGGTGCA
ACTB	TTCCTTCCTGGGCATGG	CAGGAGGAGCAATGATCTTGA
GAPDH	GTCCATGCCATCACTGCCAC	AGACGGCAGGTCAGGTCCAC

MiRNA detection primers (5'-3')

Universal reverse	GTGCAGGGTCCGAGGT
RNU6B stem loop	GTCGTATCCAGTGCAGGGTCCGAGGTATTCGCACTGGATACGA CAAAATATGGAAC
MiR-210 stem loop	GTCGTATCCAGTGCAGGGTCCGAGGTATTCGCACTGGATACGA CTCAGCC
RNU6B Fwd	GCCCCTGCGCAAGGATGAC
MiR-210 Fwd	ATGCCTGTGCGTGTGAC

APPENDIX II

Cloning primers

Name	Forward primer (5'-3')	Reverse primer (5'-3')
ALDH5A1 CDS	TATAAGCTTACCATGGCGACCTG CATTTGGCTG	AATACTCGAGCTACAAGCCCCCGT AACACAC
ALDH5A1 3' UTR	AATACTAGTACTTTCACCACAGT CAAC	TAAGAGCTCTGCTTCGTCTCCACC TAC

APPENDIX III

SDM primers (5'-3')

ALDH5A1 3' UTR MUT Fwd	CATCATTCAAATAACCGTCAAAAAACAAAAGCC
ALDH5A1 3' UTR MUT Rev	GGCTTTTGTTTTTTGACGGTTATTTGAATGATG
