# Supplementary material for: ALDH5A1/miR-210 axis plays a key role in reprogramming cellular metabolism and has a significant correlation with glioblastoma patient survival
Source: Cancer Cell Int. 2024 Jul 22;24:259. doi: 10.1186/s12935-024-03432-z (PMC11265472; doi:10.1186/s12935-024-03432-z)
Supplement: Supplementary file 2 — Supplementary Material 2. [file 12935_2024_3432_MOESM2_ESM.pdf]

Figure S1

A

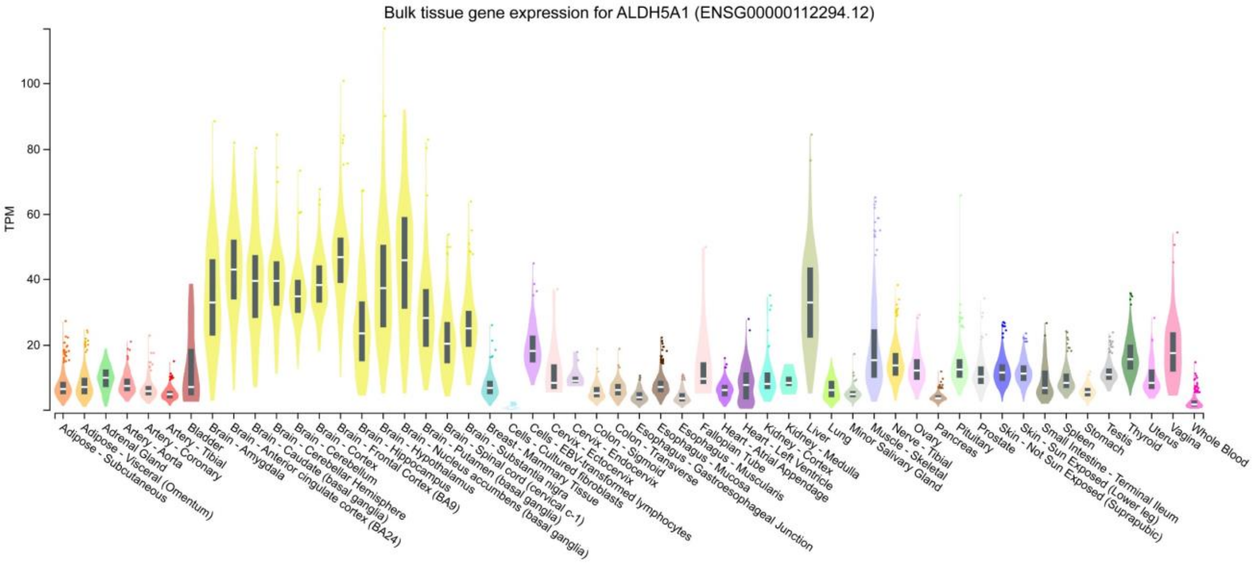

B

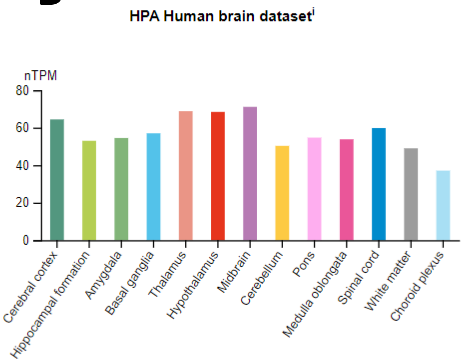

C

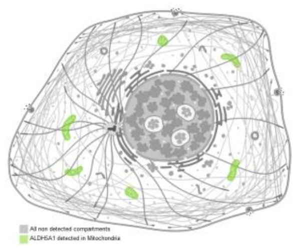

D

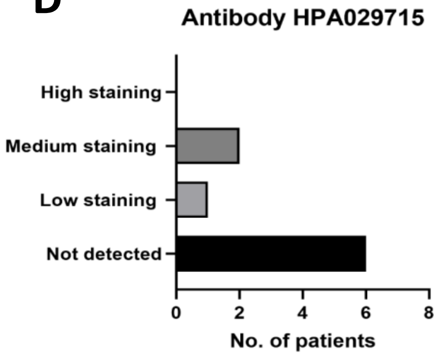

Figure S1: Tissue distribution, subcellular localization, and protein expression of ALDH5A1: (A) Bulk tissue gene expression of ALDH5A1 (GTEx portal). (B) Brain tissue gene expression of ALDH5A1 (HPA). (C) ALDH5A1 is localized to the mitochondria (HPA). (D) Quantitative representation of verified HPA029715 anti-ALDH5A1 antibody staining of glioma tissue samples (HPA). Kaplan Meier survival curve of high and low ALDH5A1 expressing GBM patients in TCGA GBM (E) and Vital (F).

Figure S2

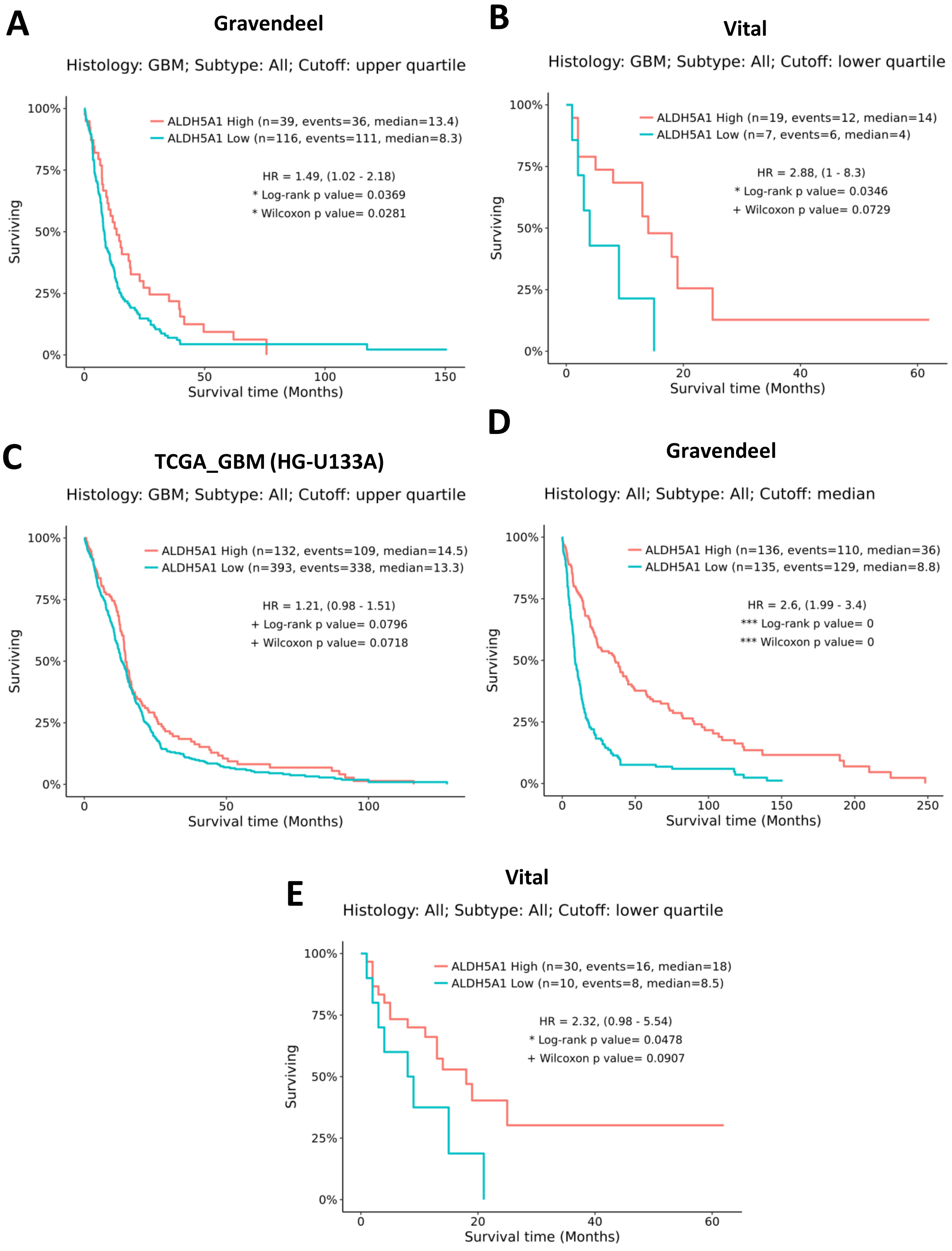

Figure S2: ALDH5A1 is associated with favorable patient survival: Kaplan Meier survival curve of high and low ALDH5A1 expressing patients in Gravendeel_GBM (A), Vital_GBM (B), TCGA GBM (C), Gravendeel_Glioma (D), and Vital_Glioma (E) datasets. Data was downloaded from GlioVis web portal.

Figure S3

A

	Predicted consequential pairing of target region (top) and miRNA (bottom)	Site type	Context++ score	Context++ score percentile	Weighted context++ score	Conserved branch length	P_{CT}	Predicted relative K_D
Position 1263-1270 of ALDH5A1 3' UTR<br>hsa-miR-210-3p	5' ...CUCAUCAUUCAAAUAACGCACAA...<br>3' AGUCGGCGACAGUGUGCGUGUC	8mer	-0.53	97	-0.28	0.029	< 0.1	-5.362

B

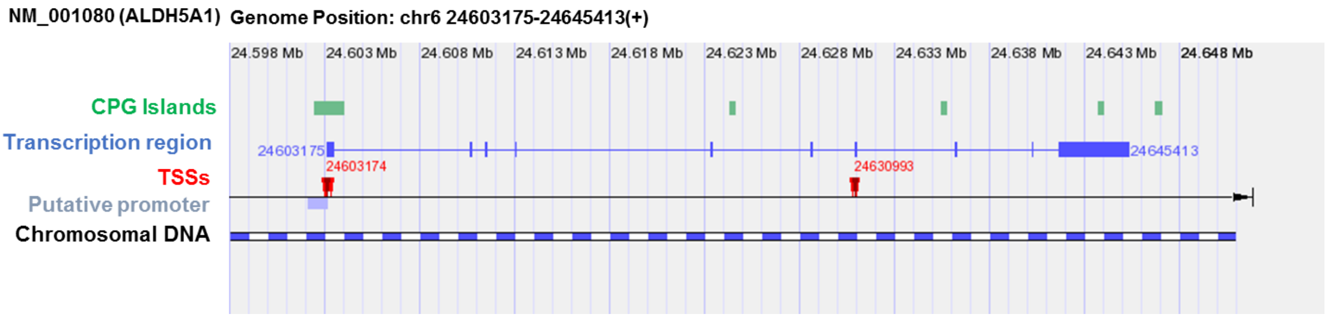

C

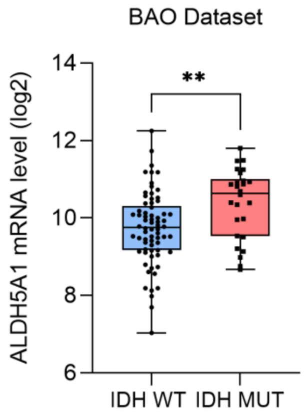

D

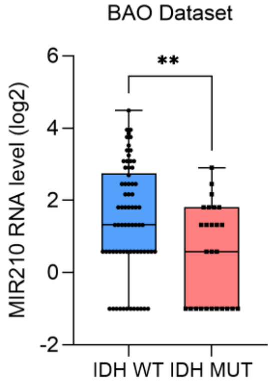

E

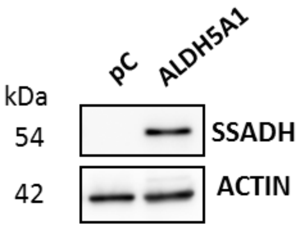

Figure S3: (A) miR-210 has a canonical 8-mer type binding site in the 3' UTR of ALDH5A1 transcript (TargetScanHuman). (B) ALDH5A1 promoter has a CpG island (DBCAT). (C) MiR-210 is downregulated in IDH mutant glioma patients of Bao dataset. (D) ALDH5A1 is upregulated in IDH mutant glioma patients of Bao dataset. (E) Validation of ALDH5A1 overexpression by immunoblotting.
